# ER-lysosome lipid transfer protein VPS13C/PARK23 prevents aberrant mtDNA-dependent STING signaling

**DOI:** 10.1083/jcb.202106046

**Published:** 2022-06-03

**Authors:** William Hancock-Cerutti, Zheng Wu, Peng Xu, Narayana Yadavalli, Marianna Leonzino, Arun Kumar Tharkeshwar, Shawn M. Ferguson, Gerald S. Shadel, Pietro De Camilli

**Affiliations:** 1 Department of Neuroscience, Yale University School of Medicine, New Haven, CT; 2 Department of Cell Biology, Yale University School of Medicine, New Haven, CT; 3 Interdepartmental Neuroscience Program, Yale School of Medicine, New Haven, CT; 4 MD/PhD Program, Yale School of Medicine, New Haven, CT; 5 Howard Hughes Medical Institute, Chevy Chase, MD; 6 Department of Genetics, Yale School of Medicine, New Haven, CT; 7 Salk Institute for Biological Studies, La Jolla, CA; 8 Program in Cellular Neuroscience, Neurodegeneration and Repair, Yale University School of Medicine, New Haven, CT; 9 Kavli Institute for Neuroscience, Yale University School of Medicine, New Haven, CT; 10 Aligning Science Across Parkinson’s (ASAP) Collaborative Research Network, Chevy Chase, MD

## Abstract

Mutations in VPS13C cause early-onset, autosomal recessive Parkinson’s disease (PD). We have established that VPS13C encodes a lipid transfer protein localized to contact sites between the ER and late endosomes/lysosomes. In the current study, we demonstrate that depleting VPS13C in HeLa cells causes an accumulation of lysosomes with an altered lipid profile, including an accumulation of di-22:6-BMP, a biomarker of the PD-associated leucine-rich repeat kinase 2 (LRRK2) G2019S mutation. In addition, the DNA-sensing cGAS-STING pathway, which was recently implicated in PD pathogenesis, is activated in these cells. This activation results from a combination of elevated mitochondrial DNA in the cytosol and a defect in the degradation of activated STING, a lysosome-dependent process. These results suggest a link between ER-lysosome lipid transfer and innate immune activation in a model human cell line and place VPS13C in pathways relevant to PD pathogenesis.

## Teaser

Hancock-Cerutti et al. demonstrate that deletion of the autosomal recessive Parkinson’s disease (PD) gene VPS13C in a model cell line causes significant perturbations of lysosomal lipid composition and leads to activation of the cGAS-STING pathway, with potential implications for PD pathogenesis.

## Introduction

Genetic studies have revealed many genes whose mutations cause or increase the risk of Parkinson’s disease (PD). Elucidating the functions of these genes, and the mechanisms by which their mutations cause PD, may provide insights into general PD pathophysiology and yield new therapeutic strategies. Several of these genes have been implicated in mitochondrial function (Malpartida et al., 2021), while many others play a role in the endolysosomal system (Abeliovich and Gitler, 2016; Vidyadhara et al., 2019). The extent of physiological and pathological cross talk between these two organelle systems is a topic of increasing interest (Hughes et al., 2020; Kim et al., 2021; Yambire et al., 2019).

One of the genes whose mutations are responsible for familial early onset PD is VPS13C (Darvish et al., 2018; Lesage et al., 2016; Schormair et al., 2018). The VPS13C locus was also identified in multiple PD genome-wide association studies (Nalls et al., 2019). Additionally, loss-of-function mutations in VPS13C genes were detected in dementia with Lewy bodies, peculiar protein aggregates enriched in α-synuclein and characteristic of PD (Smolders et al., 2021). The VPS13 gene family encodes lipid transfer proteins that localize to a variety of distinct contact sites between membranous organelles (Ugur et al., 2020). These proteins are thought to function as bridges that allow phospholipids to traverse the aqueous cytosolic environment between bilayers through a hydrophobic groove that runs along their length (Kumar et al., 2018; Li et al., 2020; Guillen-Samander et al., 2021; Cai et al., 2022).

Initial studies of VPS13C focused on a potential role in mitochondrial physiology (Lesage et al., 2016), as at the time studies of the single yeast Vps13 protein had suggested a role of this protein in the transport of lipids to these organelles (Lang et al., 2015). Such studies reported the presence of VPS13C in mitochondria-associated membrane fractions and showed that VPS13C knockdown causes mitochondrial dysfunction (Lesage et al., 2016). This seemed to be consistent with evidence for a major role of defects in mitochondrial clearance in some familial forms of PD (Pickrell and Youle, 2015). However, subsequent studies showed that while VPS13A (Kumar et al., 2018; Yeshaw et al., 2019) and VPS13D (Guillen-Samander et al., 2021) localize to contact sites between the ER and mitochondria, VPS13C localizes instead to contact sites between the ER and late endosomes/lysosomes (Kumar et al., 2018). The localization of VPS13C at endolysosomes, but not mitochondria, was also supported by proximity labeling experiments (Go et al., 2021; Liu et al., 2018), although genetic evidence for an impact of VPS13C on lysosome properties is still missing. A role of VPS13C in the endolysosomal system is not at odds with the established genetic links to PD, given that many PD genes function in the endolysosomal system (Abeliovich and Gitler, 2016). How defects in this system promote PD, however, remains to be understood.

Recent studies have implicated activation of the innate immune response in PD pathogenesis. Specifically, it has been reported that defective mitochondrial clearance in mice with Parkin and PINK1 loss-of-function mutations subjected to additional mitochondrial stressors may result in mtDNA leakage into the cytosol, leading to activation of the cyclic GMP–AMP synthase (cGAS)-stimulator of interferon genes (STING) pathway (Sliter et al., 2018). Such activation, in turn, induces the transcription of IFN-stimulated genes (ISGs) and an NF-κB–mediated inflammatory response (Motwani et al., 2019). While cGAS and STING are primarily expressed in nonneuronal cells in brain, including microglia and astrocytes (Saunders et al., 2018), there is growing evidence that some other genes involved in neurodegenerative diseases, including PD, are also expressed primarily in nonneuronal cells, including immune cells (Cook et al., 2017). Interestingly, activation of the cGAS-STING pathway due to elevated cytosolic mtDNA was observed in bone marrow–derived macrophages (BMDMs) and mouse embryonic fibroblasts (MEFs) lacking LRRK2 (Weindel et al., 2020), a PD gene associated with lysosomes (Bonet-Ponce et al., 2020). Moreover, absence of C9orf72, an amyotrophic lateral sclerosis gene and a component of a signaling complex associated with lysosomes (Amick et al., 2016), results in hyperresponsiveness to activators of STING, likely owing to impaired degradation of STING in lysosomes (McCauley et al., 2020). As multiple groups have shown that activation of STING, an ER resident protein, triggers its transport from the ER to lysosomes, where it is degraded, defective lysosomal function may delay clearance of activated STING (Gonugunta et al., 2017; Gui et al.,2019). These previous studies raise the possibility that an interplay of mitochondrial defects (such as mtDNA leakage) and lysosomal defects (such as impaired STING degradation) may synergize in the activation of the innate immune response, leading to neuroinflammation in some neurodegenerative diseases.

Intriguingly, the single yeast Vps13 gene is required for both mitochondrial integrity (Lang et al., 2015; Park et al., 2016) and proper function of the endolysosomal system (Brickner and Fuller, 1997). Moreover, this yeast protein was identified in a genetic screen for mutations that cause the escape of mtDNA to the nucleus, hence its alias yeast mitochondrial escape 3 (YME3; Thorsness and Fox, 1993). Follow-up studies of another YME gene, the ATP-dependent mitochondrial metalloprotease YME1, revealed that escape of mtDNA required degradation of mitochondrial compartments in the vacuole, the yeast equivalent of the lysosome (Campbell and Thorsness, 1998). Moreover, recent studies of mammalian YME1L demonstrate that loss-of-function results in mtDNA leakage and activation of the cGAS-STING pathway (Sprenger et al., 2021). All these findings prompted us to explore a potential activation of the cGAS-STING pathway by mtDNA in a VPS13C^KO^ cell line and its potential relationship to lysosome dysfunction.

## Results

### Loss of VPS13C results in perturbation of lysosomal homeostasis

**Figure 1.**
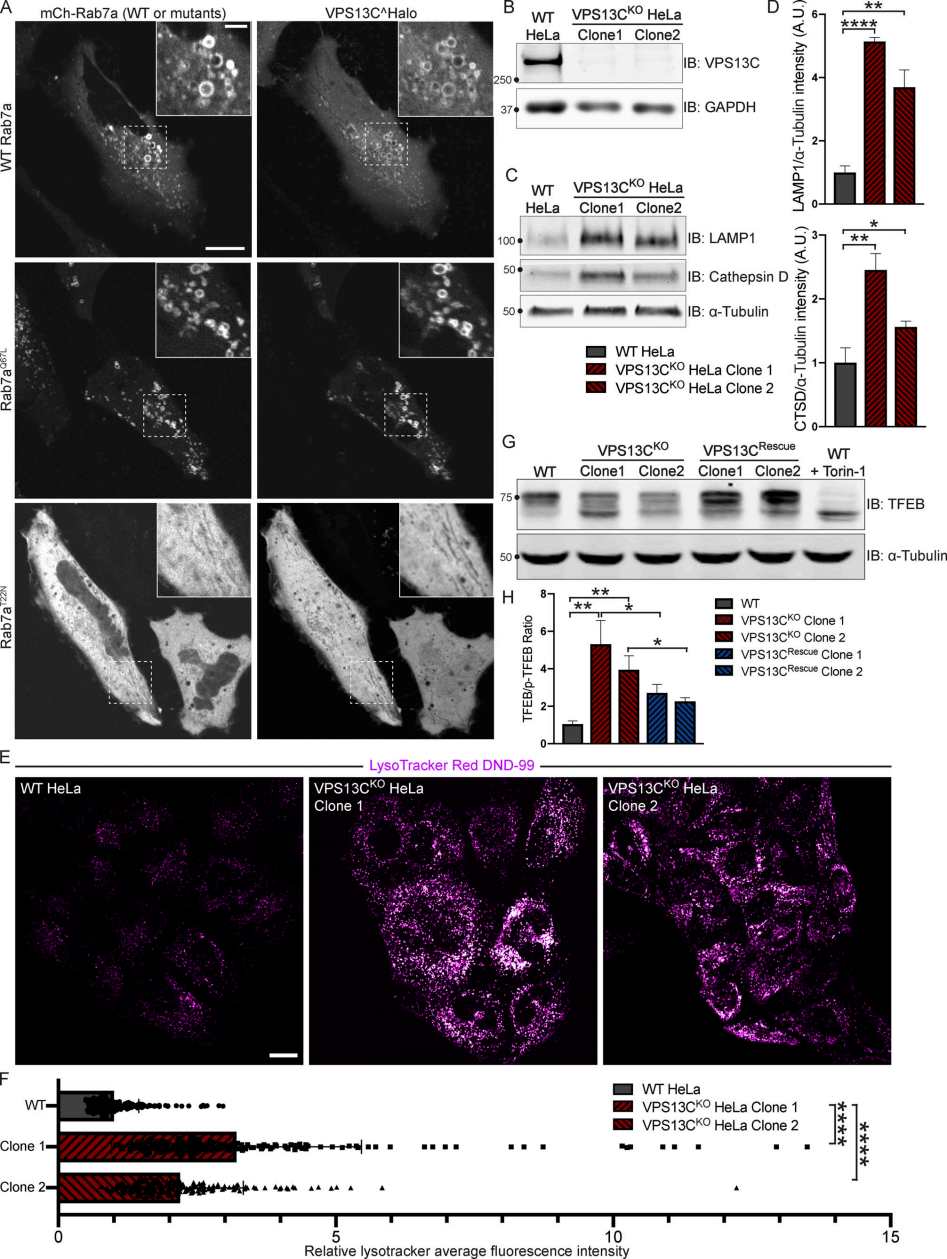
**VPS13C is a Rab7-binding protein implicated in maintaining lysosomal homeostasis. (A)** Live HeLa cells expressing full-length VPS13C^Halo with WT mCherry-Rab7a (top row), constitutively active mCherry-Rab7a^Q67L^ (middle row), or mCherry-Rab7a^T22N^ (bottom row). **(B)** IB of VPS13C in WT HeLa cells and two individual clonal cell lines after CRISPR-Cas9–mediated KO of VPS13C. **(C and D)** IB of Lamp1 and Cathepsin D in WT and VPS13C^KO^ cells (C), quantified in D. α-Tubulin was used as a loading control. *n* = 3 biological replicates. **(E and F)** Live WT and VPS13C^KO^ HeLa cells stained with LysoTracker Red DND-99 (50 nM; E), quantified in F; *n* = 3 biological replicates, >182 cells per cell line. **(G)** IB of TFEB in WT, VPS13C^KO^, and VPS13C^Rescue^ HeLa cells. As a positive control, WT cells were treated with 1 μM Torin-1 for 1 h. α-Tubulin was used as a loading control. Images from the same blot as Fig. S1 E. **(H)** Quantification of the ratio of unphosphorylated (lower band) to phosphorylated (upper band) TFEB from G. *n* = 3 biological replicates. Scale bars, 20 μm. Inset scale bars, 5 μm. *, P < 0.05; **, P < 0.01; ****, P < 0.0001. Data were compared using two-sided *t* tests. Error bars represent ±SD. Source data associated with this figure can be found at https://doi.org/10.5281/zenodo.6416363. Source data are available for this figure: SourceData F1.

**Figure S1.**
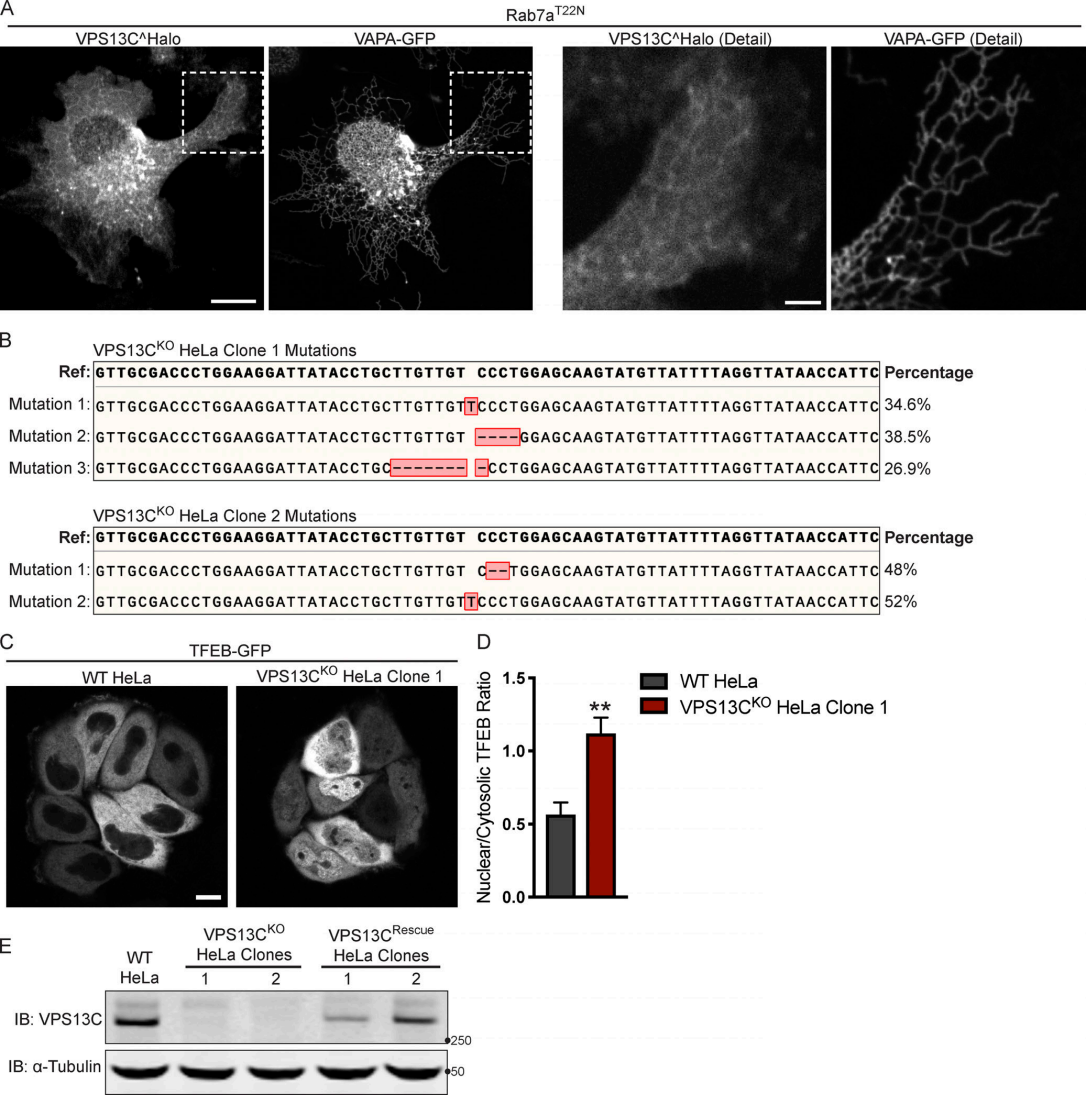
**Mutations in the VPS13C locus in VPS13C**^**KO**^ **clones 1 and 2. (A)** Live HeLa cells expressing full-length VPS13C^Halo with VAPA-GFP and mCherry-Rab7a^T22N^ (not depicted). Enlarged images of boxed areas shown to the right. **(B)** Percentage abundance of each mutated allele of 48 bacterial colonies sequenced. The HeLa cell genome is known to be aneuploid. **(C)** Live WT and VPS13C^KO^ (clone 1) HeLa cells expressing TFEB-GFP. **(D)** Quantification of nuclear to cytosolic GFP intensity from C, *n* = 175 cells across three biological replicates. **(E)** IB showing lack of VPS13C band in VPS13C^KO^ cells and return of band in repaired VPS13C^Rescue^ clones. Images from the same blot as Fig. 1 G. Scale bars, 20 μm. Inset scale bars, 5 μm. **, P < 0.01. Data were compared using a two-sided *t* test. Error bars represent ±SD. Source data associated with this figure can be found at https://doi.org/10.5281/zenodo.6416363. Source data are available for this figure: SourceData FS1.

We have previously shown that VPS13C localizes to contact sites between the ER and late endosomes/lysosomes positive for the GTPase Rab7 (Kumar et al., 2018), a marker of these organelles (Gillingham et al., 2014). Moreover, VPS13C was a hit in a high-throughput screen for interactors of Rab7a (Gillingham et al., 2019). Not only have we confirmed that VPS13C localizes to organelles positive for overexpressed Rab7a or constitutively active Rab7a^Q67L^ in HeLa cells, but we have also demonstrated that coexpression of VPS13C with a dominant-negative mutant Rab7a (Rab7a^T22N^), which cannot localize to late endosomes/lysosomes, causes VPS13C to have a diffuse cytosolic distribution (Fig. 1 A). These findings demonstrate a key role of Rab7 in the recruitment of VPS13C to late endosomes/lysosomes. In this experiment, dispersal of VPS13C to the cytosol, rather than its accumulation in the ER, the other major VPS13C-binding organelle, may reflect insufficient levels of VAP, its ER binding partner. (Kumar et al., 2018) We thus coexpressed VPS13C with VAPA and Rab7a^T22N^ (Fig. S1 A) and observed accumulation of VPS13C dispersed throughout the ER, although with significant cytosolic background. As these experiments corroborate the idea that contacts between the ER and endolysosomes are a main site of action of VPS13C, we investigated whether the absence of VPS13C has an impact on lysosomal parameters.

We generated two independent VPS13C^KO^ HeLa cell lines using CRISPR-Cas9 genome editing and confirmed indel mutations leading to premature stop codons by genomic sequencing (Fig. S1 B). Loss of protein expression was validated by immunoblotting (IB; Fig. 1 B). Both VPS13C^KO^ clones had significantly elevated levels of the lysosomal membrane protein LAMP1 and the luminal protease Cathepsin D, as assessed by Western blotting (Fig. 1, C and D). Moreover, imaging assays revealed an increased lysotracker signal (Fig. 1, E and F), further supporting an increase in lysosome abundance and showing that these lysosomes have an acidic lumen.

Transcription factor EB (TFEB) has been identified as a master regulator of lysosomal biogenesis (Sardiello et al., 2009). Under basal conditions, mTORC1-dependent phosphorylation of TFEB causes TFEB to be retained in the cytoplasm (Roczniak-Ferguson et al., 2012). However, it is dephosphorylated and translocates to the nucleus in response to a range of lysosomal stresses. Given the increase in lysosomal proteins and lysotracker fluorescence in the VPS13C^KO^ cells, we investigated whether TFEB was dephosphorylated compared with WT cells. We found that the ratio of dephosphorylated (lower band) to phosphorylated (upper band) TFEB was indeed increased in the VPS13C^KO^ cells (Fig. 1, G and H). As a positive control for TFEB dephosphorylation, WT cells were treated with the mTORC inhibitor Torin-1 (Fig. 1 G, rightmost lane). The occurrence of an increased pool of dephosphorylated TFEB in VPS13C^KO^ cells correlated with an increase in the nuclear localization of a TFEB-GFP fusion protein (Fig. S1, C and D).

To assess the specificity of this effect, we “rescued” VPS13C expression in the VPS13C^KO^ clones by using CRISPR-Cas9–mediated homology directed repair (HDR; VPS13C^Rescue^ cell lines; Fig. S1 E) and found that TFEB phosphorylation was correspondingly rescued (Fig. 1, G and H).

**Figure S2.**
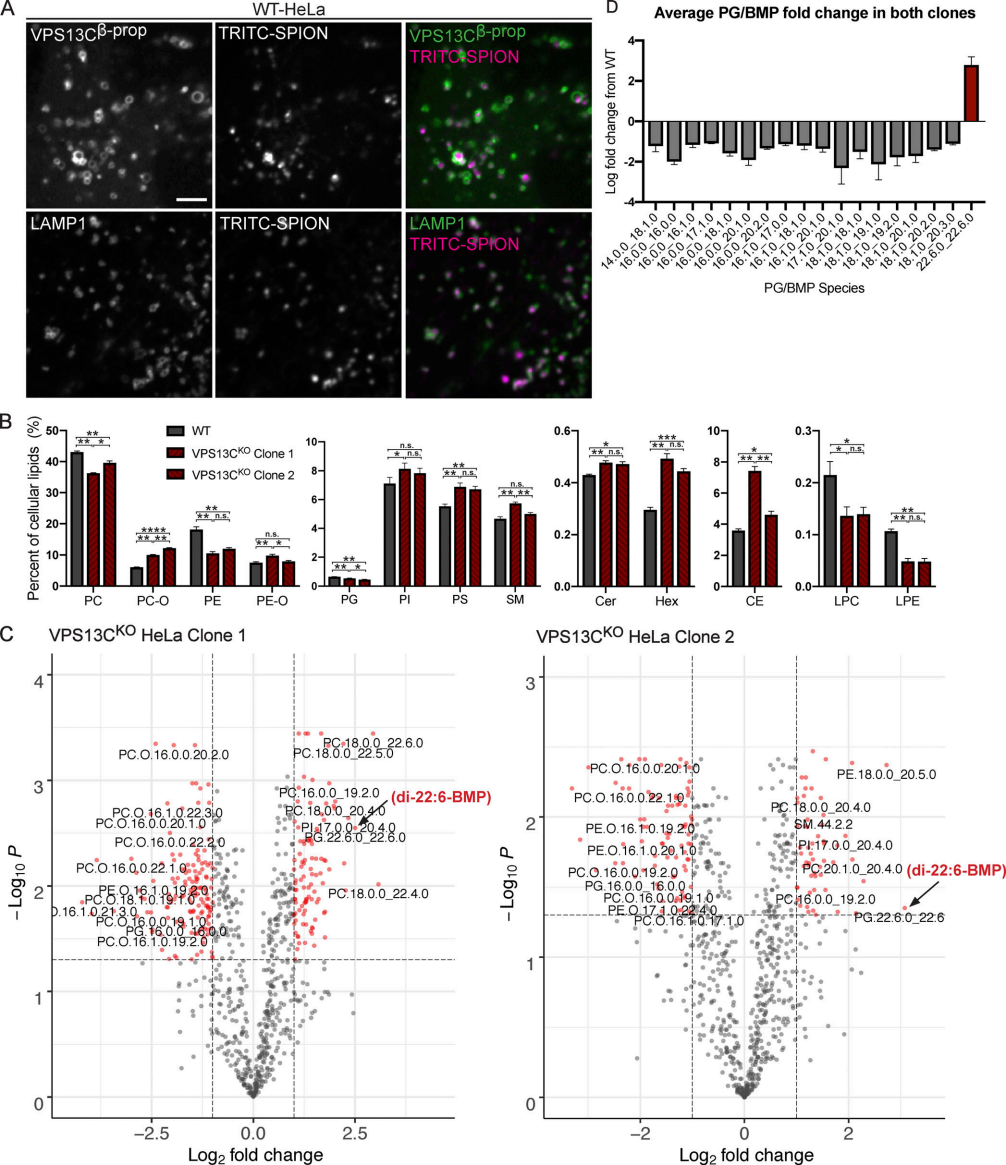
**Lipidomics of whole-cell and purified lysosomes. (A)** Colocalization of TRITC-labeled SPIONs with overexpressed GFP-VPS13C^βprop^ (top row) or LAMP1-GFP (bottom row) after 4-h pulse and 15-h chase. Scale bar, 5 μm. **(B)** Percentages of lipid classes in WT and VPS13C^KO^ whole-cell lysate normalized to total measured cell lipid content. *n* = 3 biological replicates. **(C)** Volcano plots of individual lipid species in VPS13C^KO^ purified lysosomes. Species that surpass the q-value and fold-change thresholds are shown as red dots. Lipid species labels are centered under corresponding dot. Arrows show PG.22.6.0_22.6.0/di-22:6-BMP. **(D)** Barplot showing the average fold-change of PG/BMP species in both clones. *n* = 3 biological replicates. Only species with q values < 0.05 are shown. For lipidomic data, *, q < 0.05; **, q < 0.01; ***, q < 0.001. For all other data, *, P < 0.05; **, P < 0.01; ***, P < 0.001; ****, P < 0.0001 compared with WT control. Lipidomic data in C were compared using FDR. All other data were compared using two-sided *t* tests. Error bars represent ±SD. Source data associated with this figure can be found at https://doi.org/10.5281/zenodo.6416363.

**Figure 2.**
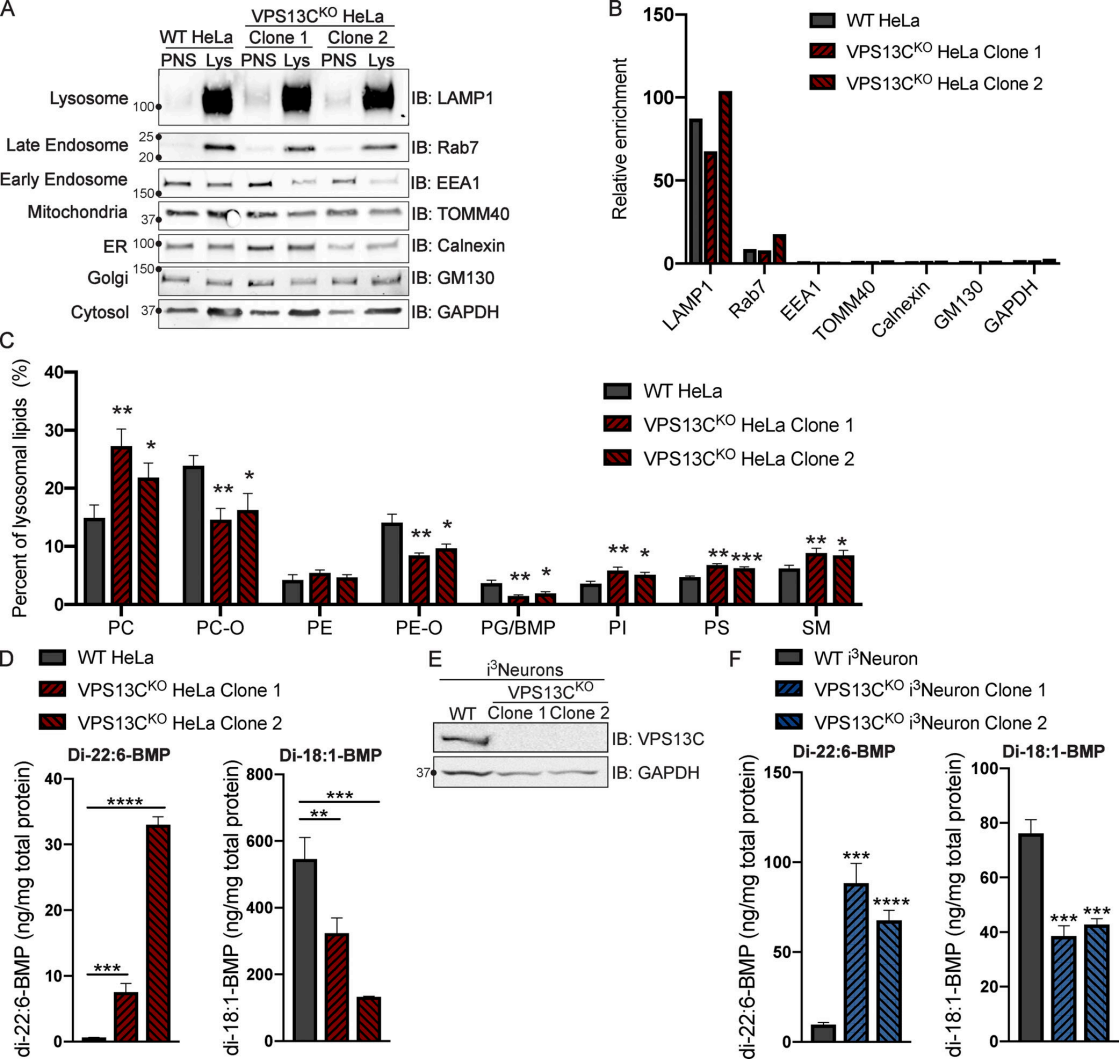
**Loss of VPS13C results in altered lysosomal lipid composition. (A)** IB showing abundance of organelle markers in postnuclear supernatant (PNS) and lysosomal fractions (Lys) from WT and VPS13C^KO^ HeLa cells. Equal amounts of total protein were loaded in each lane. Note the striking enrichment of LAMP1 in lysosomal fractions. **(B)** Quantification of band intensities from A normalized to GAPDH to show relative enrichment. **(C)** Percentages of phospho- and sphingolipid classes in WT and VPS13C^KO^ lysosomal fractions normalized to total measured lysosomal lipid content. *n* = 4 biological replicates. **(D)** Concentrations of di-22:6- and di-18:1-BMP normalized to total protein in WT and VPS13C^KO^ HeLa total cell lysate. *n* = 3 biological replicates. **(E)** IB showing loss of VPS13C protein expression in two clonal VPS13C^KO^ i^3^Neuron lines after 14-d differentiation. GAPDH was used as a loading control. **(F)** Concentrations of di-22:6- and di-18:1-BMP normalized to total protein in WT and VPS13C^KO^ i^3^Neuron (day 14) total cell lysate. *n* = 3 biological replicates. For lipidomic data, *, q < 0.05; **, q < 0.01; ***, q < 0.001. For all other data, *, P < 0.05; **, P < 0.01; ***, P < 0.001; ****, P < 0.0001 compared with WT control. Lipidomic data in C were compared using FDR. All other data were compared using two-sided *t* tests. Error bars represent ±SD. Source data associated with this figure can be found at https://doi.org/10.5281/zenodo.6416363.

Given the putative role of VPS13C as a lipid transfer protein, we next examined the impact of the absence of VPS13C on the lysosome lipidome. To isolate lysosomes, we pulsed (4 h) cells with dextran-coated superparamagnetic iron-oxide nanoparticles (SPIONs), which were taken up through bulk-endocytosis and trafficked to the endolysosomal compartment (Tharkeshwar et al., 2020; Tharkeshwar et al., 2017). Imaging of a fluorescently tagged version of these nanoparticles at 15 h after the pulse confirmed their trafficking to vesicular structures that were positive for both LAMP1 and a transfected construct containing the β-propeller region of VPS13C (VPS13C^β-prop^), i.e., the Rab7-binding region of VPS13C (Fig. S2 A). This observation confirmed the accumulation of the nanoparticles in a VPS13C-relevant compartment. Cell lysis and purification of nanoparticles-enriched lysosomes using a magnetic column yielded >67-fold enrichment of the integral lysosomal membrane protein LAMP1 relative to the control protein GAPDH, as well as enrichment of the late-endosomal marker Rab7, a peripheral membrane protein (Fig. 2, A and B).

Shotgun mass spectrometry (MS)-based lipidomic analysis of the major lipid classes in the lysosomal fractions revealed substantial differences between VPS13C^KO^ and controls on a percent molar basis (specific lipid class versus total lipids) that were consistent in both VPS13C^KO^ clones. There were increases in phosphatidylcholine (PC), phosphatidylserine (PS), phosphatidylinositol (PI), and sphingomyelin (SM), as well as a decrease in phosphatidylglycerol (PG). PG measurement may include bis(monoacylglycerol)phosphate (BMP), as PG and BMP are structural isomers that were not distinguished by this analysis (Fig. 2 C). In addition, ether-lipid forms of both phosphatidylcholine (PC-O) and phosphatidylethanolamine (PE-O) were significantly reduced in the lysosomes of both VPS13C^KO^ clones (Fig. 2 C). Ether lipid synthesis involves the peroxisome and the ER (Jimenez-Rojo and Riezman, 2019). Importantly, there was no decrease in these lipids, and even a slight increase, in the total cell lipidome (Fig. S2 B), suggesting that biosynthesis is intact and that the decrease of ether lipids in lysosomes may result from a defect in trafficking to the lysosome. The whole-cell lipidome also revealed increases in ceramide, hexosylceramide, and cholesteryl ester, as well as decreases in lysoPC (LPC) and lysoPE (LPE; Fig. S2 B). Collectively, these findings reveal a perturbation of lysosomal lipid homeostasis in VPS13C^KO^ cells.

### Enhanced levels of di-22:6-BMP in VPS13C^KO^ cells

We next analyzed individual lipid species in control and VPS13C^KO^ lysosomes. Among the 1,161 species measured, we found that 123 of them were significantly altered in both VPS13C^KO^ clones relative to controls (Fig. S2 C). In agreement with the class-level decreases, most of the downregulated hits were PC-O and PE-O species. The upregulated hits comprised a variety of classes including PC, PI, PE, and SM, many containing polyunsaturated fatty acid tails including arachidonic acid (20:4), eicosapentaenoic acid (20:5), docosapentaenoic acid (22:5), and docosahexaenoic acid (22:6). The most highly increased lipid species in one of the VPS13C^KO^ clones and the third highest in the other one, was PG(22.6_22.6; Fig. S2 C, black arrowhead), which, as stated above, could not be distinguished from its structural isomer di-22:6-BMP. As BMPs (also referred to as LBPA) are specific to the endolysosomal system (Gruenberg, 2020), we suspected that the majority of the species reported as PG(22.6_22.6) was actually di-22:6-BMP. This was intriguing, as di-22:6-BMP has been established as a biomarker for a number of neurodegenerative diseases, including Niemann Pick type C (Liu et al., 2014) and, more recently, LRRK2 G2019S mutation status (Alcalay et al., 2020). The increase of di-22:6-BMP in whole-cell lysate was confirmed by a quantitative MS assay specifically designed to assess this lipid (Fig. 2 D). Moreover, di-18:1-BMP, which appears to be the most abundant species in HeLa cells and is a major species in most human tissues (Showalter et al., 2020) was decreased (Fig. 2 D). This is consistent with our findings that total PG/BMP species are decreased by approximately half in VPS13C^KO^ Hela lysosomes, revealing an overall decrease in total BMP but a specific increase in di-22:6-BMP (Figs. 2 C and S2 D).

To investigate this phenotype in a neuronal cell type, we knocked out VPS13C using CRISPR-Cas9 in induced pluripotent stem cells (iPSCs) with a stably integrated NGN2 ORF under a doxycycline-inducible promoter (i^3^Neurons; Fig. 2 E; Fernandopulle et al., 2018; Wang et al., 2017). We differentiated these cells into neurons for 14 d according to established protocols (Fernandopulle et al., 2018; Gowrishankar et al., 2021). As in the VPS13C^KO^ HeLa cells, di-22:6-BMP was increased in two separate VPS13C^KO^ i^3^Neuron clones (Fig. 2 F), whereas di-18:1-BMP was decreased. While the significance of the specific increase in di-22:6-BMP and decrease of total BMP remains unclear, this finding is consistent with alteration of lysosomal function across multiple neurodegenerative conditions.

Total BMP is also reduced in certain subtypes of neuronal ceroid lipofuscinosis (Hobert and Dawson, 2007) and is altered by knockout (KO) of Progranulin, a lysosomal protein whose loss-of-function mutation causes both neuronal ceroid lipofuscinosis (homozygous) and frontotemporal dementia (heterozygous; Logan et al., 2021).

### Activation of the cGAS-STING pathway in VPS13C^KO^ HeLa cells

**Figure 3.**
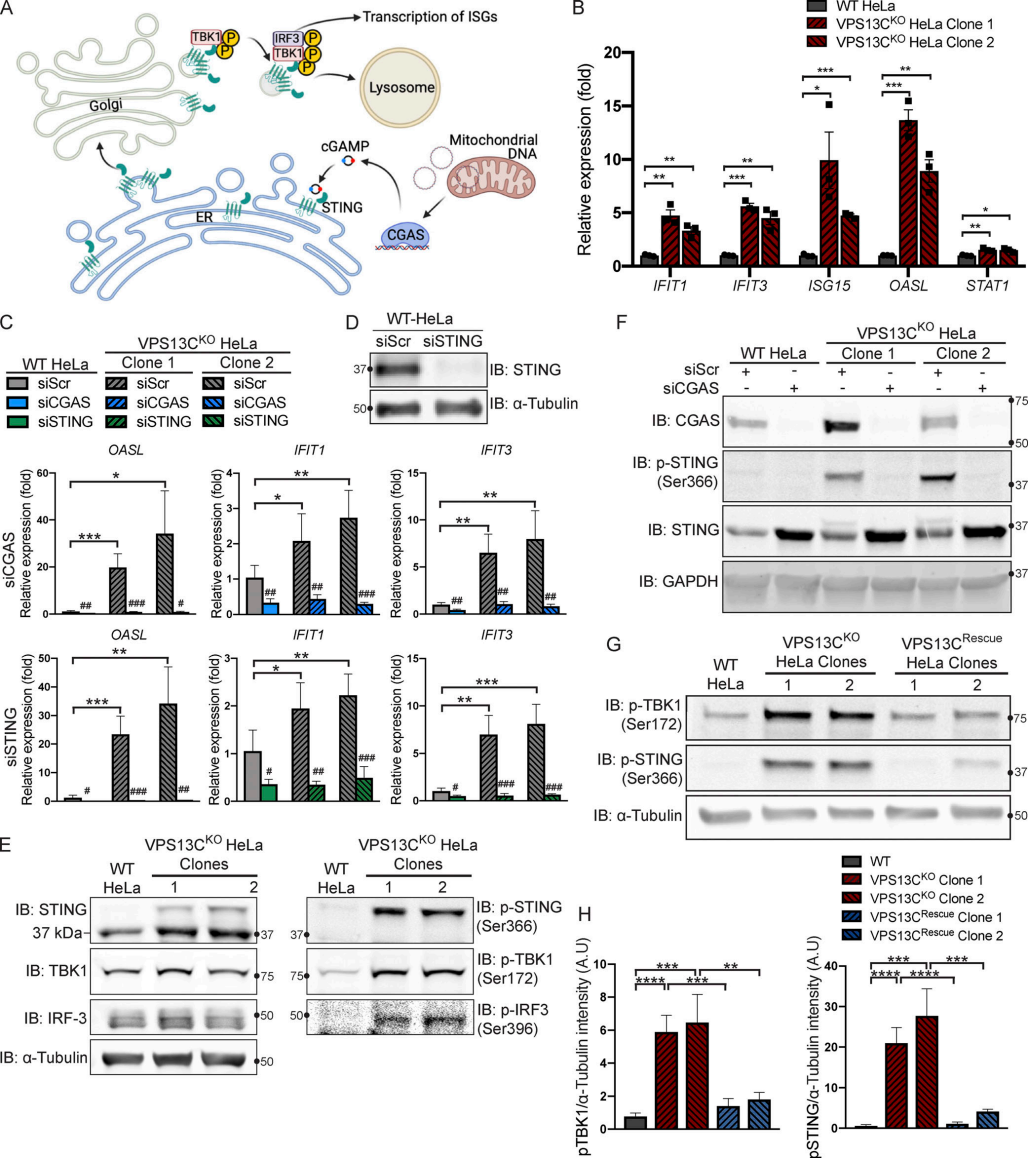
**Loss of VPS13C results in activation of the cGAS-STING pathway. (A)** Cartoon schematic of cGAS-STING signaling pathway and STING trafficking through the Golgi to lysosomes for degradation. **(B)** qPCR of five ISG transcripts (*IFIT1*, *IFIT3*, *ISG15*, *OASL*, and *STAT1*) shows increased expression in VPS13C^KO^ HeLa cells. *n* = 3 biological replicates. Created with BioRender.com. **(C)** qPCR of three ISG transcripts after treatment with siRNA against cGAS (top row) or STING (bottom row). *n* = 4 biological replicates. **(D)** IB showing efficiency of STING depletion after treatment with anti-STING siRNA. **(E)** IB showing increased levels of phosphorylated STING, TBK1, and IRF3, indicating activation of the cGAS-STING pathway. Note that the upper band in lanes 2 and 3 of the anti-STING blot corresponds to p-STING (lanes 2 and 3 of the p-STING blot). **(F)** Treatment of siRNA against cGAS significantly depletes cGAS and also returns p-STING to WT levels in the VPS13C^KO^ clones. cGAS knockdown also causes an increase in total STING levels in both WT and VPS13C^KO^ cells. **(G and H)** P-TBK1 and p-STING are returned toward WT levels in VPS13C^Rescue^ clones (G), quantified in H. *n* = 3 biological replicates. *, P < 0.05; **, P < 0.01; ***, P < 0.001; ****, P < 0.0001. #, P < 0.05; ##, P < 0.01; ###, P < 0.001 in siCGAS or siSTING compared with siScr-treated cells. Data were compared using two-sided *t* tests. Error bars represent ±SD. Source data associated with this figure can be found at https://doi.org/10.5281/zenodo.6416363. Source data are available for this figure: SourceData F3.

Having defined an impact of the lack of VPS13C on lysosomal homeostasis, we next addressed the hypothesis that the absence of VPS13C in HeLa cells could result in an activation of the cGAS-STING pathway (Fig. 3 A). First, as a readout of potential cGAS-STING activation, we analyzed a subset of ISGs (IFIT1, IFIT3, ISG15, OASL, and STAT1) by quantitative PCR (qPCR) and saw increased expression in both VPS13C^KO^ HeLa clones (Fig. 3 B). This increase was no longer observed when cGAS (Fig. 3 F) or STING (Fig. 3 D) was knocked down by siRNA (Fig. 3 C). Knockdown of cGAS or STING also decreased ISG expression in our WT HeLa cells, suggesting some basal level of STING signaling in these cells.

Upon binding to cGAMP, activated STING undergoes a conformational change, oligomerizes, traffics through the Golgi, and recruits the kinase TBK1, which phosphorylates STING at ser366 as well as itself at ser172 (Liu et al., 2015; Shang et al., 2019; Zhang et al., 2019). Phosphorylated STING subsequently recruits IRF3, which is phosphorylated and activated by TBK1 (Liu et al., 2015) and undergoes translocation to the nucleus to induce ISG expression (Fig. A; Lin et al., 1998). Again, consistent with cGAS-STING activation, phosphorylated forms of STING, TBK1, and IRF3 were all significantly elevated in VPS13C^KO^ HeLa cells (Fig. 3 E). Total STING levels were also slightly increased (Fig. 3 E). Additionally, siRNA knockdown of cGAS abolished STING-Ser366 phosphorylation in VPS13C^KO^ HeLa cells, while global levels of STING were slightly increased consistent with lower basal degradation in the absence of cGAS (Fig. 3 F).

We next investigated whether activation of cGAS-STING could be returned to baseline in the VPS13C^Rescue^ cells. In clones in which VPS13C protein expression had been successfully restored, phospho-STING and phospho-TBK1 were restored to WT or near-WT levels (Fig. 3, G and H).

### Role of mtDNA escape in cGAS-STING activation in VPS13C^KO^ HeLa cells

**Figure S3.**
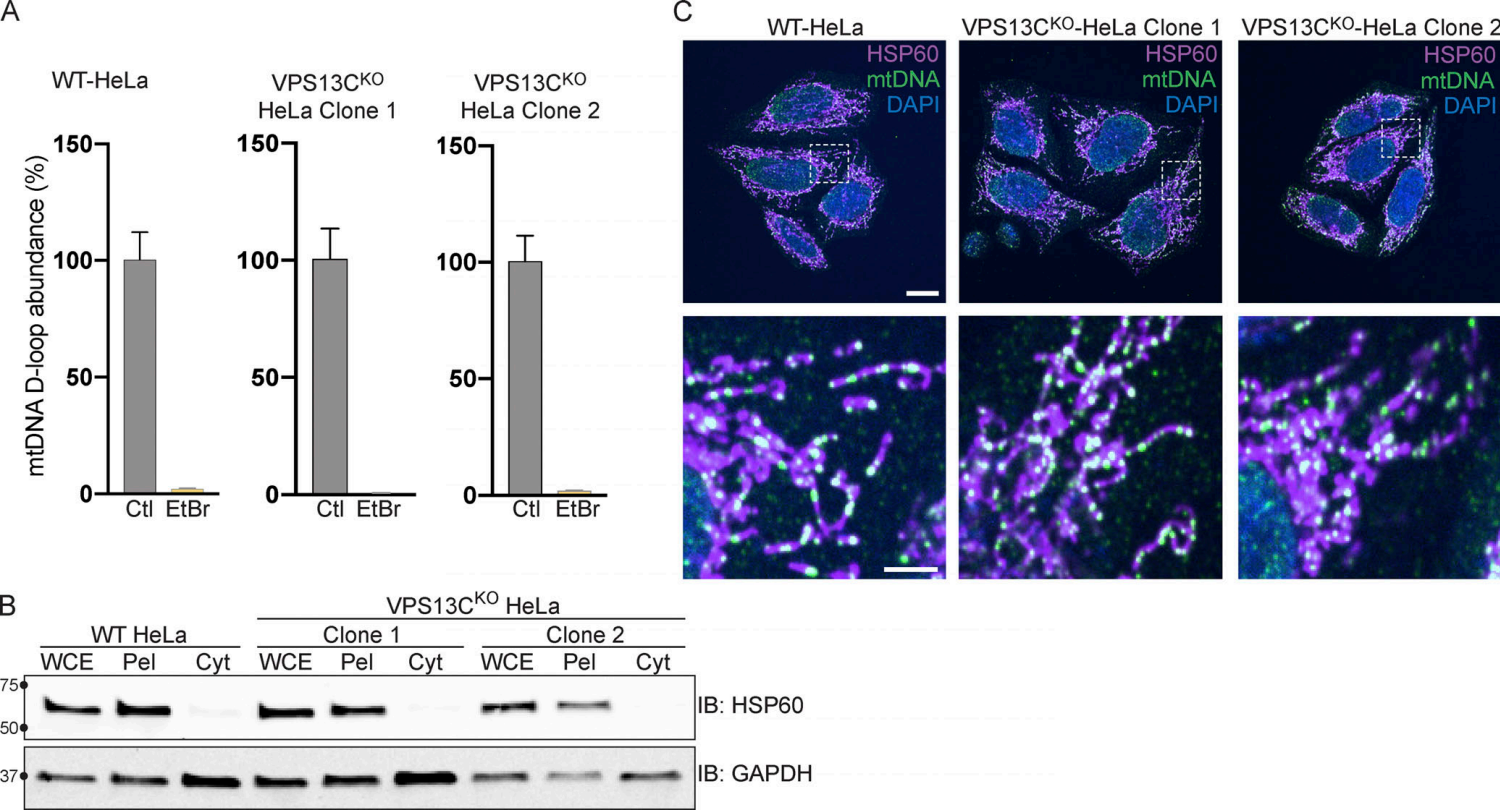
**Control experiments for data shown in Fig. 4 and normal mtDNA nucleoid morphology in VPS13C^KO^ cells. (A)** qPCR of a D-Loop mtDNA amplicon relative to the nuclear gene hB2M shows efficient depletion of mtDNA after treatment with EtBr. The controls reflect each of the cell lines (WT, VPS13C^KO^ clone 1, and VPS13C^KO^ clone 2) grown in normal media. *n* = 3 biological replicates. **(B)** IB showing that mitochondrial marker HSP60 is present in the whole cell extract (WCE) and pellet (Pel) but absent in the cytosolic fraction (Cyt), while the cytosolic marker GAPDH is present in all fractions. **(C)** Immunofluorescence showing that mitochondria (magenta) and mtDNA nucleoids (green) have grossly normal morphology in VPS13C^KO^ HeLa cells. Scale bar, 20 μm. Inset scale bars, 5 μm. Error bars represent ±SD. Source data associated with this figure can be found at https://doi.org/10.5281/zenodo.6416363. Source data are available for this figure: SourceData FS3.

**Figure 4.**
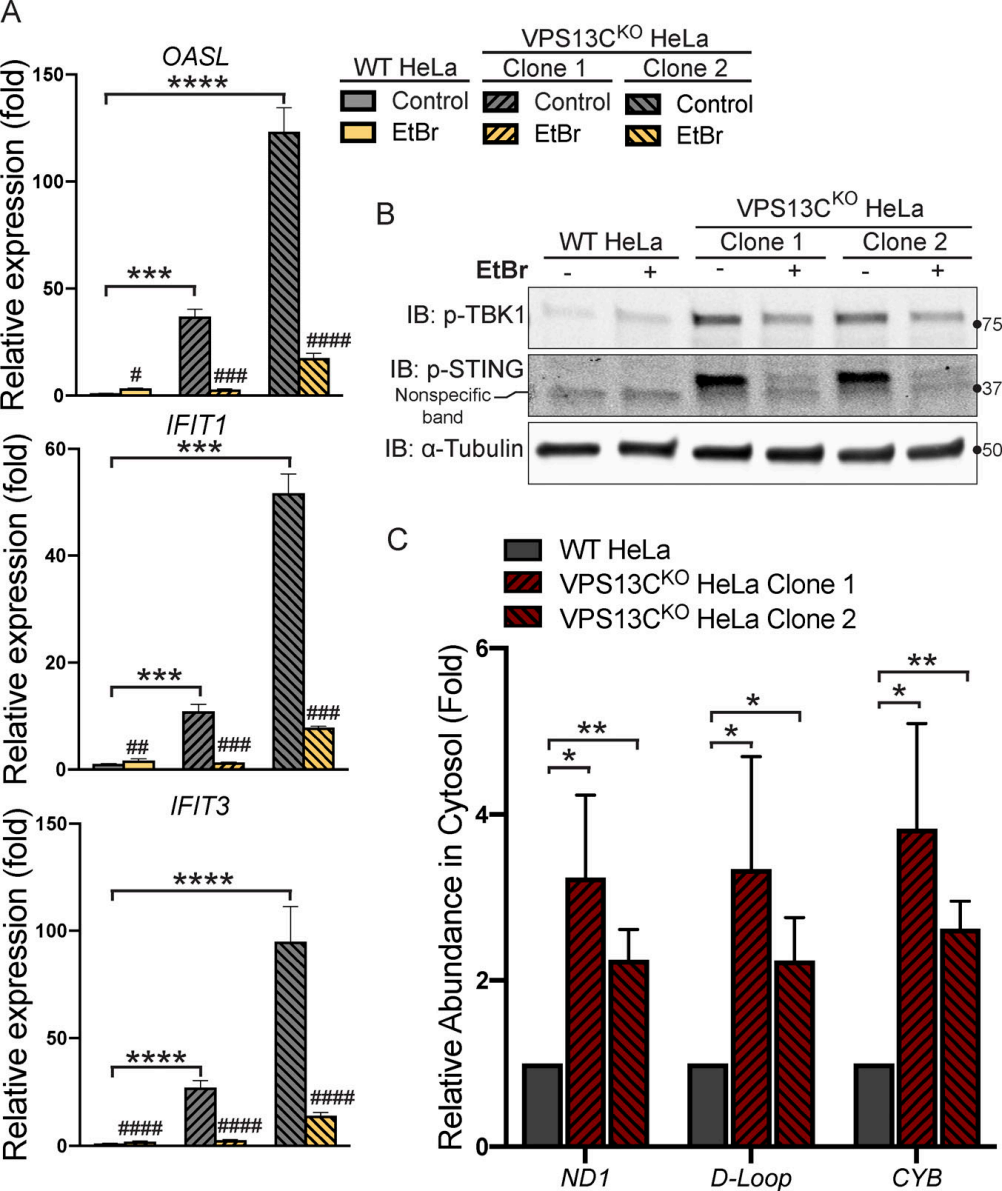
**Activation of the cGAS-STING pathway in VPS13C**^**KO**^ **cells is dependent on increased cytosolic mtDNA. (A)** qPCR of three ISG transcripts shows that depletion of mtDNA with EtBr reduces ISG levels in VPS13C^KO^ cells to or near WT levels. *n* = 3 biological replicates. **(B)** p-TBK1 and p-STING are reduced in EtBr-treated VPS13C^KO^ cells. **(C)** Levels of three mtDNA amplicons (ND1, D-Loop, and CYB) are elevated in the cytosolic fraction of VPS13C^KO^ cells detected by qPCR and normalized to nuclear gene hB2M. *n* = 3 biological replicates. *, P < 0.05; **, P < 0.01; ***, P < 0.001; ****, P < 0.0001. #, P < 0.05; ##, P < 0.01; ###, P < 0.001; #### < 0.0001 in EtBr compared with untreated cells. Data were compared using two-sided *t* tests. Error bars represent ±SD. Source data associated with this figure can be found at https://doi.org/10.5281/zenodo.6416363. Source data are available for this figure: SourceData F4.

Because mtDNA is a ligand for cGAS and hence an activator of STING (West et al., 2015), and mutations in the single yeast Vps13 protein result in escape of mtDNA (Thorsness and Fox, 1993), we considered the possible role of mtDNA leakage in the activation of STING observed in VPS13C^KO^ cells. While VPS13C localization does not suggest a direct impact of this protein on mitochondrial function, recent studies have revealed that alteration of lysosome function can indirectly impact mitochondria (Hughes et al., 2020; Kim et al., 2021; Yambire et al., 2019). Moreover, mitochondrial defects were reported in Cos7 cells upon siRNA-mediated VPS13C knockdown (Lesage et al., 2016). To assess whether STING activation in VPS13C^KO^ cells was dependent on mtDNA, we treated VPS13C^KO^ cells with ethidium bromide (EtBr) for 72 h to deplete mtDNA (Khozhukhar et al., 2018). The efficacy of this treatment was verified by qPCR of the D-loop region of mtDNA, which demonstrated a >97% depletion in mtDNA levels (Fig. S3 A). Depletion of mtDNA in VPS13C^KO^ cells reversed both the elevated expression of ISGs and the increased levels of phospho-STING and phospho-TBK1 (Fig. 4, A and B).

To determine whether excess mtDNA is present in the cytosol in VPS13C^KO^ cells, we isolated a cytosolic fraction by centrifugation and quantified levels of three different mtDNA transcripts by qPCR. We observed a two–fourfold increase in mtDNA (Fig. 4 C) in the cytosolic fractions of VPS13C^KO^ cells, in which absence of mitochondria was documented by IB for the mitochondrial marker HSP60 (Fig. S3 B). We detected no gross differences in either mitochondrial morphology or mtDNA nucleoid distribution in VPS13C^KO^ cells by immunofluorescence (Fig. S3 C). Together, these results demonstrate that increased cytosolic mtDNA is the primary cause of cGAS-STING activation in VPS13C^KO^ HeLa cells.

### Steady-state change in the localization of STING in VPS13C^KO^ HeLa cells

**Figure S4.**
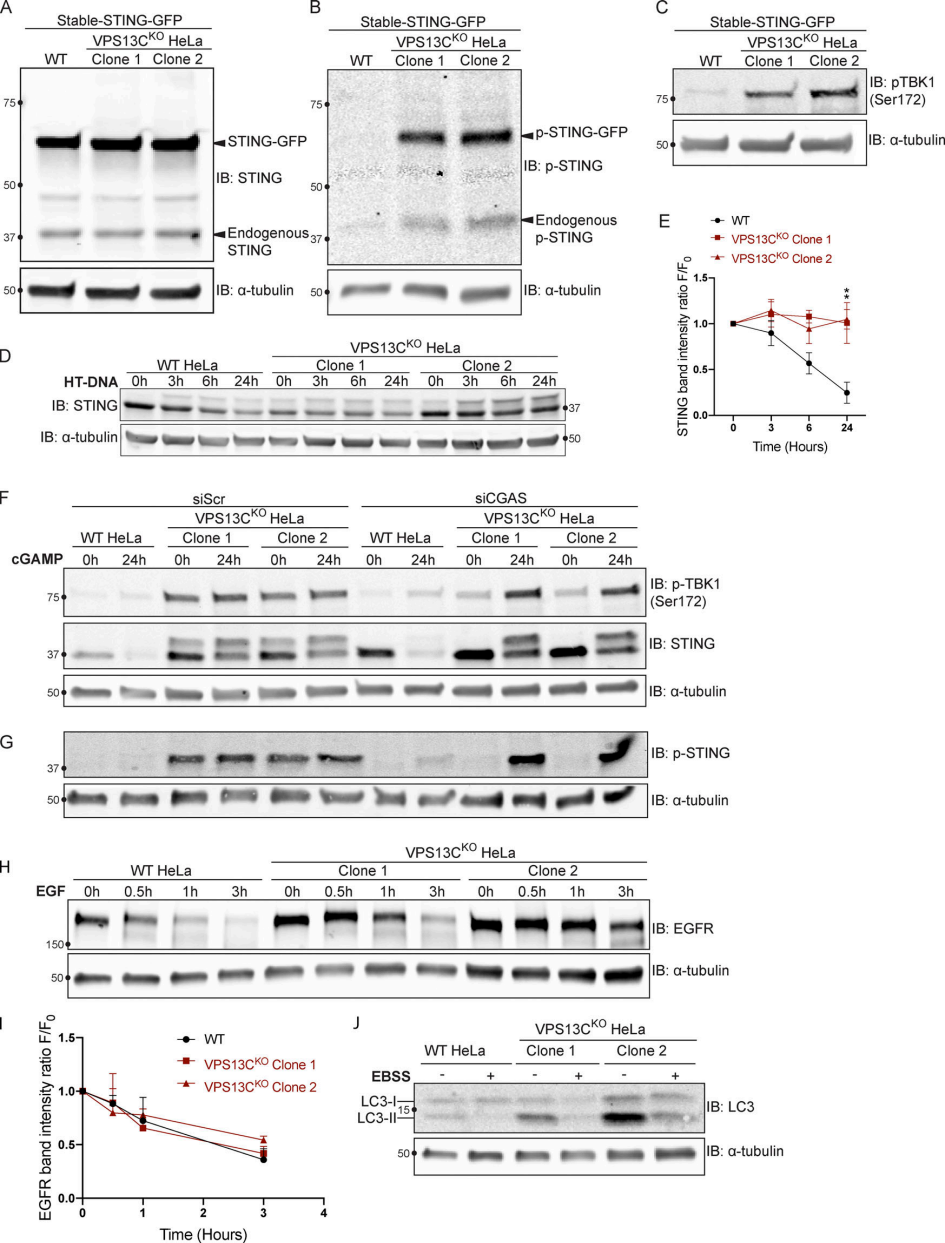
**Supplemental data for Figs. 5 and 6 with EFGR and LC3 degradation. (A)** IB showing similar levels of stable STING-GFP expression in WT and VPS13C^KO^ cells. **(B)** IB showing increased levels of p-STING in VPS13C^KO^ cells (both endogenous and STING-GFP). **(C)** IB showing increased levels of p-TBK1 in VPS13C^KO^ cells. **(D)** Time course of WT and VPS13C^KO^ cells treated with HT-DNA (500 ng/ml) for 0, 3, 6, and 24 h. **(E)** As with cGAMP treatment, STING is not significantly degraded in VPS13C^KO^ cells, quantified in E. *n* = 2 biological replicates. **(F)** Intact blot from which the data from Fig. 6, E and G, were extracted. **(G)** IB of the same samples as in F with anti p-STING antibody. **(H)** IB of EGFR in WT and VPS13C^KO^ cells treated with 100 ng/ml EGF for 0.5, 1, and 3 h. **(I)** Quantification from A of band intensity at various timepoints (F) normalized to EGFR intensity at 0 h (F_0_), showing no defect in EGFR degradation kinetics. *n* = 3 biological replicates. **(J)** IB of LC3 in WT and VPS13C^KO^ cells after 6-h starvation in Earle’s balanced salt solution (EBSS). Note that the ratio of lipidated LC3-II to LC3-I is elevated under basal conditions in VPS13C^KO^ cells, possibly downstream of STING activation, as previously reported (Fischer et al., 2020; Gui et al., 2019). **, P < 0.01. Time course data were compared using two-way ANOVA followed by FDR multiple comparisons testing. All other data were compared using two-sided *t* tests. Error bars represent ±SD. Source data associated with this figure can be found at https://doi.org/10.5281/zenodo.6416363. Source data are available for this figure: SourceData FS4.

**Figure 5.**
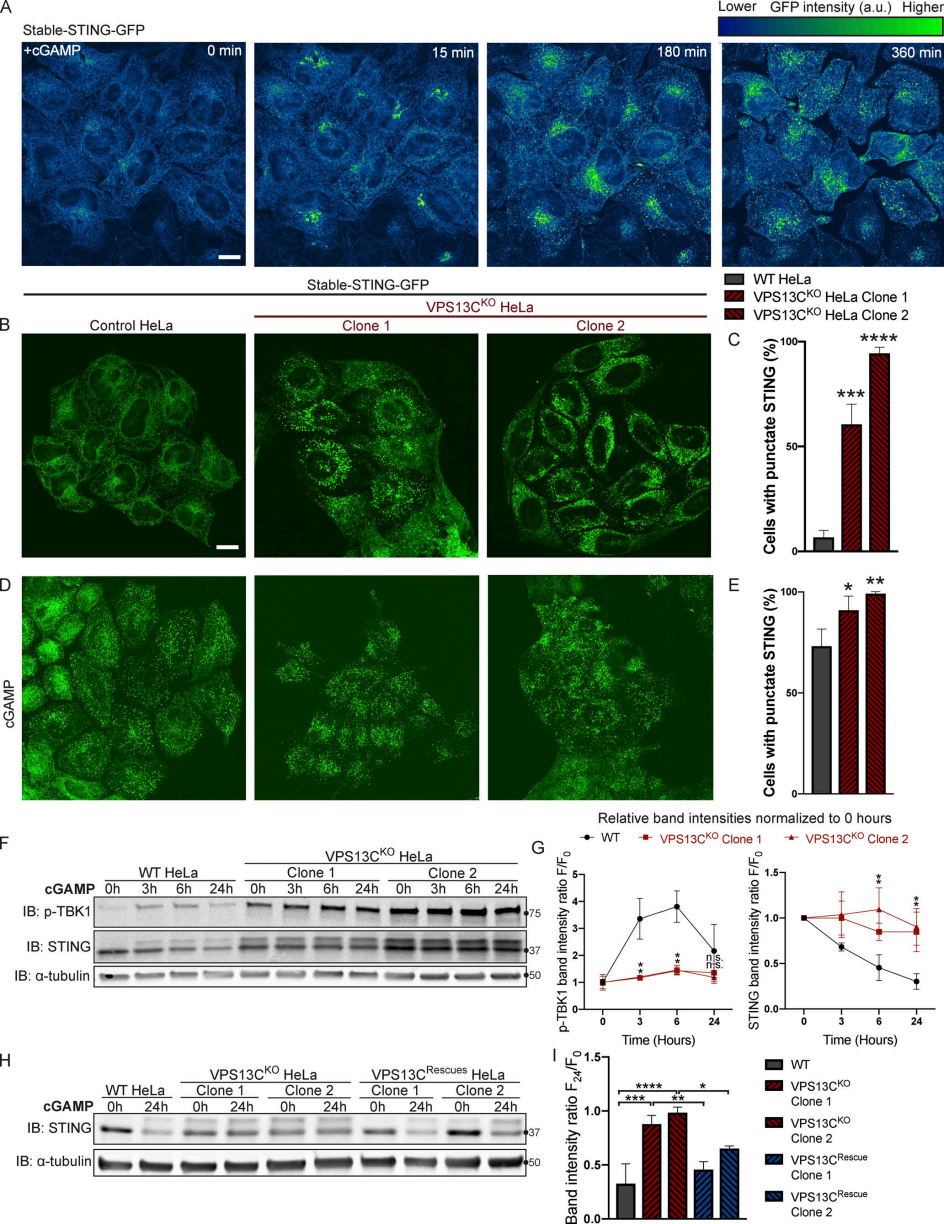
**STING is activated and translocated out of the ER at baseline in VPS13C**^**KO**^ **cells. (A)** Selected frames from time lapse of stable STING-GFP in WT HeLa cells after treatment with 50 µg/ml cGAMP (Video 1). The GFP signal is shown using the “Green Fire Blue” lookup table in ImageJ, in which lower intensities are displayed in blue and higher intensities are displayed in green, a key for which is shown at top right. STING-GFP is localized in an ER-like pattern 0 min after treatment and traffics to a Golgi-like pattern 15 min after treatment, a Golgi/vesicular pattern by 180 min after treatment, and a largely vesicular pattern by 360 min. **(B)** Under unstimulated basal conditions, STING-GFP is localized in an ER distribution in WT HeLa cells but a vesicular distribution in the majority of VPS13C^KO^ cells. **(C)** The percentage of cells with vesicular pattern is quantified in C. *n* = 3 biological replicates. **(D and E)** treatment with cGAMP had only minimal effect on the already punctate distribution of STING-GFP in VPS13C^KO^ cells, but induces a vesicular pattern in the majority of WT cells (D), quantified in E. *n* = 3 biological replicates. **(F)** In WT cells, treatment with 8 µg/ml cGAMP causes an increase in p-TBK1 at 3 and 6 h time points and a return to baseline at 24 h, along with a concomitant decrease in total STING levels over 24 h as STING is degraded. In VPS13C^KO^ cells, the same treatment fails to cause a significant increase in p-TBK1, STING upper band (phospho-STING), or decrease in total STING levels. **(G)** Band intensity of p-TBK1 and STING at each time point is quantified relative to the 0-h value for each cell line. For quantification of the STING bands, both the upper and lower band were included. All bands were normalized to the loading control. *n* = 3 biological replicates. **(H and I)** Treatment with 8 µg/ml cGAMP in VPS13C^Rescue^ cells for 24 h results in STING degradation closer to WT levels, but fails to induce significant STING degradation in VPS13C^KO^ cells (H), quantified in I. *n* = 3 biological replicates. Scale bars, 20 μM. *, P < 0.05; **, P < 0.01; ***, P < 0.001; ****, P < 0.0001 compared with WT control. cGAMP time course data were compared using two-way ANOVA followed by FDR multiple comparisons testing. All other data were compared using two-sided *t* tests. Error bars represent ±SD. Source data associated with this figure can be found at https://doi.org/10.5281/zenodo.6416363. Source data are available for this figure: SourceData F5.

The cGAMP-dependent oligomerization of STING leading to its activation also triggers its transport from the ER via the Golgi complex to lysosomes, where it is degraded leading to termination of signaling (Gonugunta et al., 2017; Gui et al., 2019). Thus, we tested whether the constitutive activation of STING observed in VPS13C^KO^ cells is accompanied by a change in its steady-state localization. As transient transfection, which involves acute introduction of plasmid DNA, can activate cGAS-STING, we generated cell lines stably expressing STING-GFP in control and VPS13C^KO^ HeLa cells via retroviral transduction. STING-GFP expression was similar in control and VPS13C^KO^ cells (Fig. S4 A), and phospho-STING (Ser366) and phospho-TBK1 (Ser172) remained elevated in the VPS13C^KO^ cells (Fig. S4, B and C). In WT cells, STING-GFP was almost exclusively localized to the ER, as expected (Fig. 5, A and B). Treatment with cGAMP, the product of cGAS that binds to STING, caused STING to concentrate in a Golgi complex–like pattern within 15 min and then to disperse throughout the cells as punctate structures, previously shown to overlap in part with lysosomes over the next 6 h (Fig. 5 A and Video 1; Gui et al., 2019). In contrast, in VPS13C^KO^ cells (not exposed to exogenous cGAMP), STING-GFP already had a predominantly punctate localization (Fig. 5, B and C) similar to the localization of STING-GFP in WT cells 12 h after cGAMP stimulation (Fig. 5, D and E).

**Video 1.** **Treatment with cGAMP induces translocation of STING-GFP from the ER, through the Golgi complex, to dispersed vesicles in WT HeLa cells.** Time-lapse of stably expressed STING-GFP in WT HeLa cells after treatment with 50 µg/ml cGAMP just before start of imaging, one frame every 15 min from 0 to 360 min. STING-GFP is localized in an ER-like pattern at 0 min after treatment and traffics to a Golgi-like pattern 15 min after treatment, a Golgi/vesicular pattern by 180 min after treatment, and a largely vesicular pattern by 360 min.

We complemented these localization studies with biochemical experiments. In WT cells, addition of herring testes (HT)-DNA to activate cGAS (Fig. S4 D), or of cGAMP to directly activate STING (Fig. 5 F), resulted in the appearance of the upper STING band (the phosphorylated form) and increased levels of phospho-TBK1, but also in the degradation of total STING over time, as reported (Gonugunta et al., 2017; Gui et al., 2019). After 24 h, total levels of STING were reduced to 25% (for HT-DNA; Fig. S4, D and E) and 30% (for cGAMP; Fig. 5, F and G) of baseline, and phospho-TBK1 also returned toward baseline (Fig. 5, F and G). In contrast, in VPS13C^KO^ cells both phospho-TBK1 and phospho-STING were already elevated at baseline (total STING was also elevated), and addition of cGAMP did not promote degradation of these proteins (Fig. 5, F and G). These differences from WT were rescued in the VPS13C^Rescue^ cells, where the VPS13C mutation had been repaired (Fig. 5, H and I). Collectively, these findings, i.e., elevated levels of both total STING and phospho-STING, and lack of its responsiveness to stimulation, raised the questions of whether the cGAS-STING pathway could still be activated by exogenous cGAMP in VPS13C^KO^ cells and whether degradation of STING might also be impaired.

### Silencing of cGAS unmasks cGAMP responsiveness of VPS13C^KO^ HeLa cells and reveals impaired STING degradation

**Figure 6.**
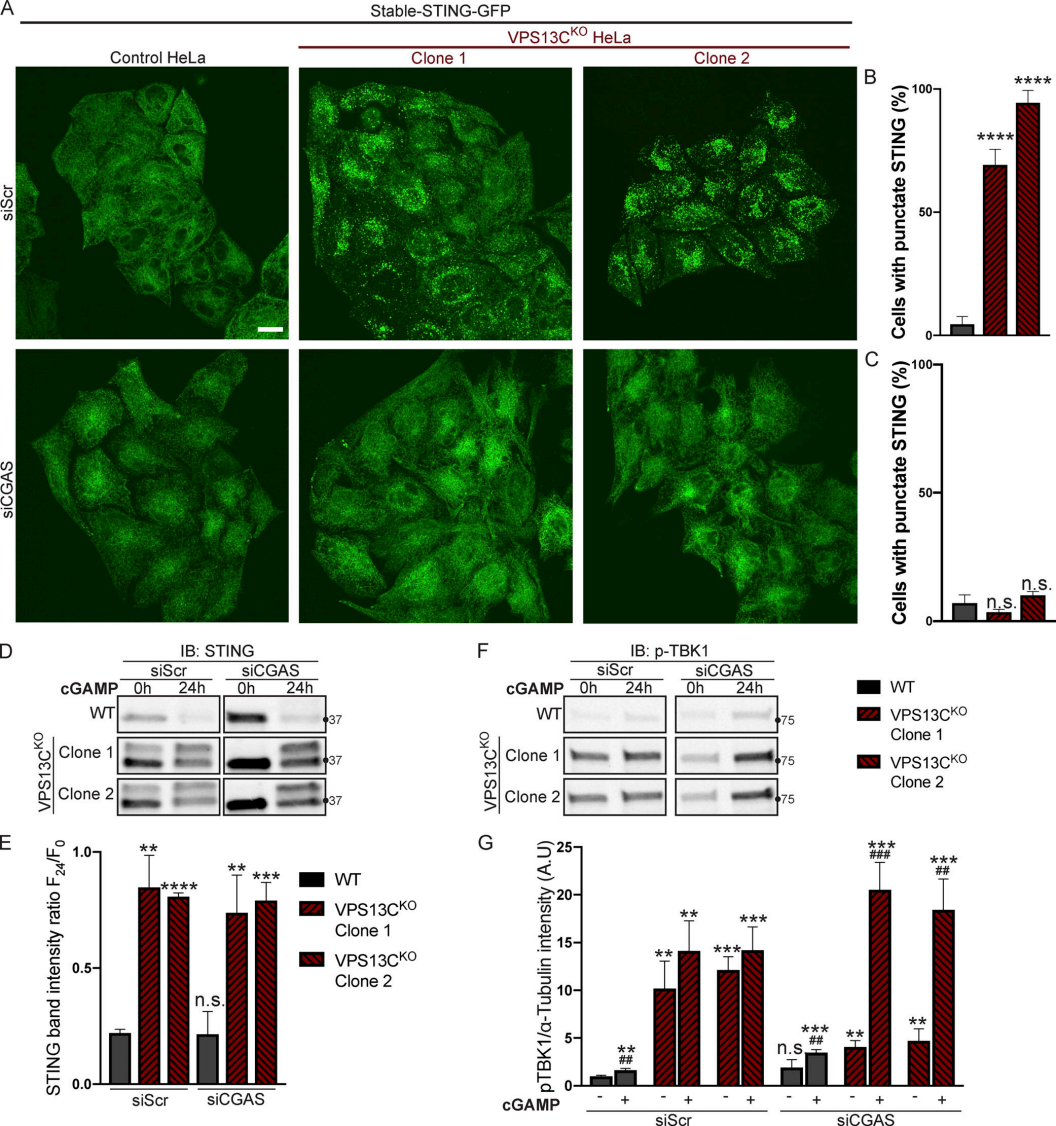
**Silencing of cGAS unmasks cGAMP responsiveness and reveals impaired STING degradation in VPS13C**^**KO**^ **cells. (A–C)** Treatment with siRNA against cGAS returns STING-GFP to an ER distribution in VPS13C^KO^ cells, similar to untreated WT cells (A), quantified in B, while siScr has no effect, quantified in C. *n* = 3 biological replicates. **(D)** IB against STING in WT and VPS13C^KO^ cells treated with either scrambled siRNA or siRNA against CGAS, followed by treatment with 8 µg/ml cGAMP for 24 h. Treatment with siRNA against CGAS returns STING to the unphosphorylated state in VPS13C^KO^ cells, rendering them responsive to cGAMP. In VPS13C^KO^ cells, the activation of STING is not followed by degradation, compared with WT cells. **(E)** Quantification of the total STING signal remaining 24 h after cGAMP treatment (F_24_) relative to 0 h (F_0_). For quantification of the STING bands, both the upper and lower band were included. All bands were normalized to the loading control. *n* = 3 biological replicates. **(F)** IB against p-TBK1 under the same conditions as E. WT cells behaved similarly in response to cGAMP treatment regardless of siScr or siCGAS pretreatment, with p-TBK1 levels returning to baseline after 24 h of cGAMP treatment, as shown by the time course of Fig. 5, F and G. In VPS13C^KO^ cells pretreated with siScr, p-TBK1 remained at baseline after 24 h of cGAMP treatment, presumably never having increased, based on the time course in Fig. 5, F and G. In VPS13C^KO^ cells pretreated with siCGAS, however, p-TBK1 was significantly elevated after 24 h of cGAMP, in accordance with the defect in STING degradation and continued STING signaling (E and F). **(G)** Quantification of p-TBK1 band intensity. All bands were normalized to the loading control. *n* = 3 biological replicates. Scale bars, 20 μM. **, P < 0.01; ***, P < 0.001; ****, P < 0.0001 compared with untreated WT cells. ##, P < 0.01; ###, P < 0.001 value at 24-h cGAMP treatment compared with corresponding 0-h cGAMP treatment. Data were compared using two-sided *t* tests. Error bars represent ±SD. Source data associated with this figure can be found at https://doi.org/10.5281/zenodo.6416363. Source data are available for this figure: SourceData F6.

To determine whether VPS13C^KO^ cells can still respond to cGAMP, they were treated with siRNA against cGAS (with scrambled siRNA as a control), to suppress basal STING activation. cGAS knockdown reverted STING-GFP to an ER localization (Fig. 6, A and C), similar to untreated WT cells, while scrambled siRNA had no effect (Fig. 6, A and B).

Biochemical experiments confirmed the restoration of responsiveness to cGAMP of VPS13C^KO^ cells. Both phospho-TBK1 (Fig. 6, F and G), and phospho-STING (Fig. S4 G), (also reflected by the upper total STING band [Fig. 6 D]) returned to unstimulated WT levels after treatment with anti-cGAS siRNA (full blot in Fig. S4 F). Upon addition of cGAMP, we observed only a very modest reduction of total STING levels at 24 h in VPS13C^KO^ cells compared with significant STING degradation in WT cells, suggesting a bona fide defect in STING degradation (Fig. 6, D and E). Furthermore, phospho-TBK1 (Fig. 6, F and G) and phospho-STING (Fig. S4 G) remained elevated in VPS13C^KO^ cells at 24 h, in agreement with sustained STING signaling.

The defect in STING degradation did not reflect an overall defect in protein degradation in lysosomes, as we found no difference in the kinetics of EGFR degradation in response to EGF stimulation between VPS13C^KO^ and WT cells (Fig. S4, H and I). Likewise, we found no difference in LC3 degradation after induction of macro autophagy by starving cells in EBSS (Fig. S4 J). Interestingly, the ratio of lipidated LC3-II to LC3-I under basal conditions was increased in the VPS13C^KO^ cells (Fig. S4 J), consistent with the reported property of activated STING to induce LC3 lipidation (Fischer et al., 2020; Gui et al., 2019).

### Vps13c^−/−^ mice do not demonstrate motor phenotypes or STING activation

**Figure S5.**
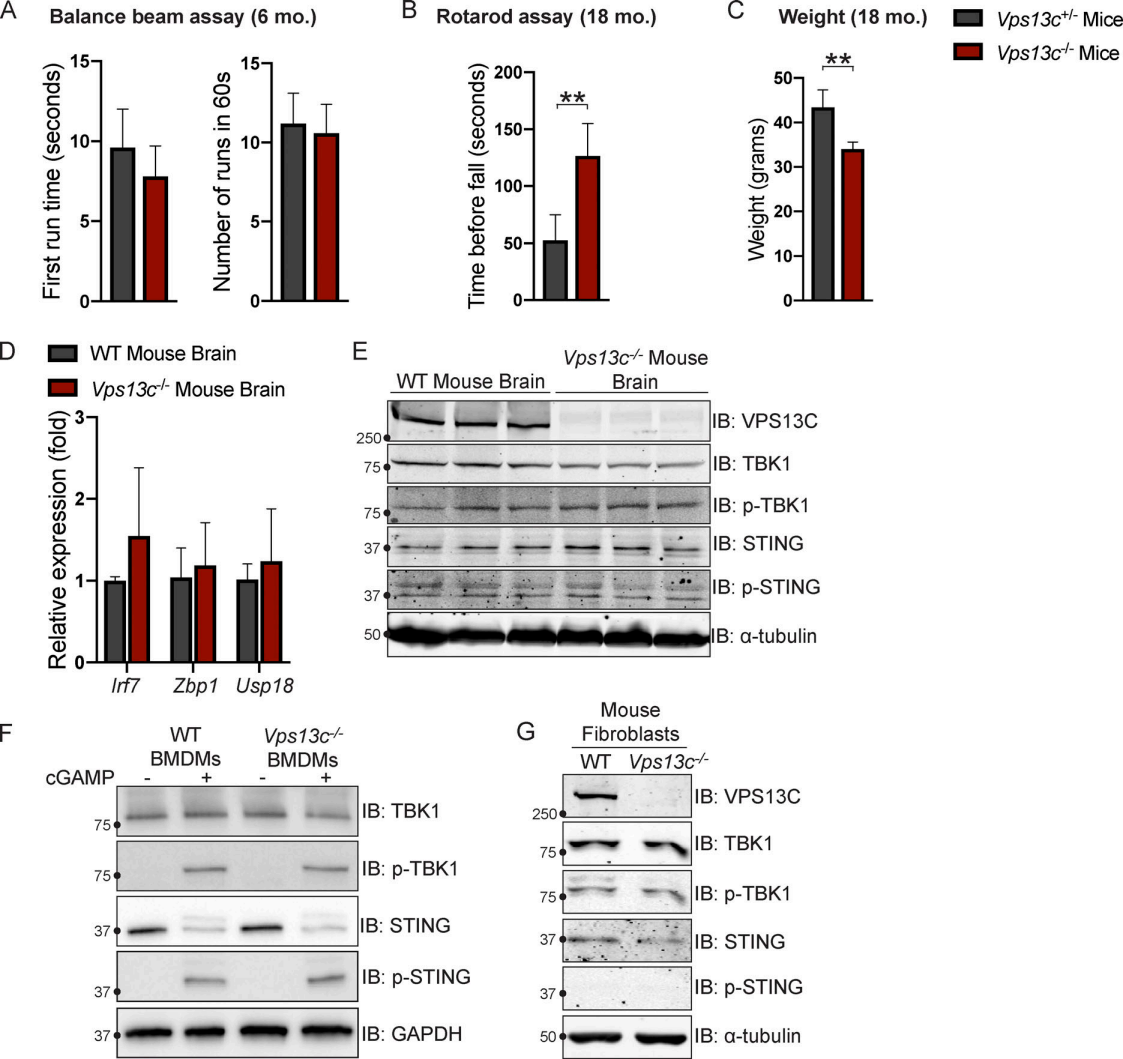
***Vps13c***^**−/−**^ **mice do not display motor deficits or STING activation. (A)** First run time (in seconds) and total number of completed runs in 60 s in the balance beam assay for *Vps13c*^−/−^ and *Vps13c*^−/+^ mice at 6 mo old. *n* = 5. **(B)** Average time until fall over four runs in the Rotarod assay for *Vps13c*^−/−^ and *Vps13c*^−/+^ mice at 18 mo old. *n* = 4. **(C)** Average weight of the mice used for the Rotarod assay in B. **(D)** qPCR of three ISG transcripts (*Irf7*, *Zbp1*, and *Usp18*) shows no significant difference between *Vps13c*^−/−^ and WT brain lysates from 1-yr-old mice. *n* = 5 biological replicates. **(E)** IB showing no significant difference in levels of phosphorylated STING and TBK1 between *Vps13c*^−/−^ and WT mouse brain lysates from 1-yr-old animals. *n* = 3 biological replicates. **(F)** IB showing no significant difference in levels of phosphorylated STING, TBK1, and IRF3 between *Vps13c*^KO^ and WT BMDMs under basal conditions and after treatment with 10 μM cGAMP for 24 h. *n* = 3 biological replicates. **(G)** IB showing no significant difference in levels of phosphorylated STING, TBK1, and IRF3 between *Vps13c*^−/−^ and WT fibroblasts. **, P < 0.01. Data was compared using a two-sided *t* test. Error bars represent ±SD. Source data associated with this figure can be found at https://doi.org/10.5281/zenodo.6416363. Source data are available for this figure: SourceData FS5.

To investigate VPS13C biology in a model animal system we used *Vps13c*^−/−^ mice. Since VPS13C-associated PD is autosomal recessive, we used either WT (*Vps13c*^WT^) or heterozygous (*Vps13c*^+/−^) animals as controls. We did not observe any obvious motor phenotypes, including hindlimb clasping, in the *Vps13c*^−/−^ mice up to 2 yr of age, nor did we observe any effect on lifespan. We assessed for motor phenotypes that had shown differences in other PD mouse models (Cao et al., 2017; Vidyadhara et al., 2022). Balance beam testing at 6 mo of age showed no difference between *Vps13c*^−/−^ mice and heterozygous controls (Fig. S5 A). Neither *Vps13c*^−/−^ mice nor heterozygous controls were able to complete the balance beam assay at 18 mo old, so we assessed them by Rotarod. Surprisingly, when subjected to Rotarod testing at 18 mo of age *Vps13c*^−/−^ mice actually performed better than *Vps13c*^+/−^ controls (Fig. S5 B). One possible explanation for this unexpected finding is that the *Vps13c*^−/−^ mice weighed significantly less than their heterozygous counterparts (Fig. S5 C), a finding which is itself of interest given that VPS13C has been shown to be associated with lipid droplets, adipogenesis, and lipolysis by multiple groups (Kumar et al., 2018; Ramseyer et al., 2018; Yang et al., 2016).

In accordance with a lack of motor phenotype, we detected no evidence of ISG upregulation using qPCR or STING/TBK1 phosphorylation by IB in mouse brain lysates from 1-yr-old *Vps13c*^−/−^ mice compared with WT controls (Fig. S5, D and E). We also did not detect differences in STING, TBK1, or IRF3 phosphorylation in BMDMs from *Vps13c*^−/−^ or WT mice, either under basal conditions or after cGAMP treatment (Fig. S5 F), nor did we detect differences in STING or TBK1 phosphorylation levels in fibroblasts derived from *Vps13c*^−/−^ or WT animals (Fig. S5 G).

## Discussion

Our results support a role of VPS13C in regulating lysosome function and show that cellular perturbations produced by the absence of VPS13C in HeLa cells result in activation of the cGAS-STING pathway. Such activation is of special interest, because VPS13C is a PD gene, and aberrant activation the cGAS-STING pathway has been implicated in PD pathogenesis (Sliter et al., 2018).

We have previously shown colocalization of VPS13C at the interface between the ER and organelles positive for Rab7, a marker of late endosomes and lysosomes. Moreover, VPS13C was identified in a screen for Rab7 effectors (Gillingham et al., 2019). Our present finding that dominant-negative Rab7 completely blocks VPS13C recruitment to lysosomes proves its major role in controlling VPS13C localization. Importantly, we show that absence of VPS13C results in alterations in lysosome homeostasis, as demonstrated by an increase in the levels of lysosome markers and by alterations of the lipid composition of purified lysosomal fractions. Increases were observed in PC, PI, PS, and SM, with decreases in ether-linked phospholipids (PC-O and PE-O) and in the majority of species of BMP, a class of lipids characteristic of multivesicular bodies and lysosomes (Gruenberg, 2020). Whether these changes are directly or indirectly related to the property of VPS13C to transport lipids remains to be explored, as do the functional implications of these changes. Of note, however, was the robust and highly specific accumulation of di-22:6-BMP in both lysosomes and total cell lysates, in spite of the overall reduction of BMP, as a specific increase in urinary di-22:6-BMP was reported to be a marker of LRRK2 G2019S mutation status (Alcalay et al., 2020). Like the absence of VPS13C, the LRRK2 G2019S mutation increases PD risk (West et al., 2005). It will be interesting to determine whether elevated di-22:6-BMP is causally linked to PD pathogenesis or is simply a marker of more general lysosome dysfunction.

Our finding that the cGAS-STING signaling pathway is activated in VPS13C^KO^ HeLa cells adds to evidence for a potential involvement of this pathway in PD pathogenesis, as first suggested by studies of PINK1 and Parkin mouse models (Sliter et al., 2018). We were somewhat surprised to see robust STING signaling in our HeLa cells, as it was previously reported that the STING pathway in HeLa cells is subject to significant oncogene suppression (Lau et al., 2015). However, we found that in even in our control HeLa cells, from which the VPS13C clones were derived, STING could be activated in response to either cGAMP (Fig. 5, A, F, G, and H) or HT-DNA (Fig. S4 D). We cannot fully discount that the phenotypes we observe in our HeLa cells are influenced to some degree by accompanying changes in oncogene expression or activity. Yet, our rescue of the STING VPS13C KO phenotypes by repair of the VPS13C mutation clearly shows their dependence on VPS13C. So far, we have not observed STING activation in tissues of *Vps13c*^−/−^ mice; however these mice also do not show obvious neurological deficit and thus may not constitute a useful model for human VPS13C-associated PD. We also note that even in the case of PINK1/Parkin-KO mice (Sliter et al., 2018) the mice required additional perturbation from either exhaustive exercise or additional mtDNA mutation burden to manifest STING activation/inflammation. The role of activation of this pathway in PD, and more generally in neurodegenerative diseases, is an area of intense investigation (McCauley et al., 2020; Sliter et al., 2018; Weindel et al., 2020; Yu et al., 2020).

Our results in HeLa cells suggest that the increase of STING signaling may result both from a leakage of mtDNA, which in turn would activate cGAS and thus generation of the STING ligand cGAMP, and from a delayed degradation of activated STING in VPS13C^KO^ cells. The mechanisms underlying this delayed degradation remain elusive. Presumably, STING must remain facing the cytosol (and not be internalized in the lysosomal lumen) to continue activating TBK1 and IRF3. A defective or incomplete fusion of STING-positive vesicles with late endosomes/lysosomes or a defect in the incorporation of STING into intraluminal vesicles of late endosomes are potential mechanisms. We speculate that alterations to the lysosomal lipidome may be responsible for these defects. Interestingly, defective lysosomal degradation of activated STING, leading to higher levels of innate immune signaling, was reported in cells deficient in C9orf72, another neurodegeneration gene associated with lysosomes (McCauley et al., 2020).

An attractive unifying scenario is that alteration of lysosome function is the most proximal consequence of VPS13C depletion, and that such alteration is upstream of mitochondrial dysfunction and STING activation in our cell model system. More specifically, defective lysosomal function may be the primary event leading to mtDNA leakage. A similar scenario may apply to STING activation by the absence of LRRK2, another PD protein implicated in lysosome function (Bonet-Ponce et al., 2020; Weindel et al., 2020). Indeed, it is now appreciated that genetic or pharmacologic disruption of lysosome function can lead to mitochondria dysfunction in a number of contexts (Hughes et al., 2020; Kim et al., 2021; Yambire et al., 2019). This cross talk may be mediated by soluble factors (Hughes et al., 2020; Yambire et al., 2019) or by direct mitochondria lysosome contacts (Wong et al., 2018). It is also possible that leakage of mtDNA may occur during mitophagy by defective lysosomes. While mtDNA can escape directly from mitochondria into the cytosol (Riley and Tait, 2020; West and Shadel, 2017), as in the case of TFAM deficiency (West et al., 2015) or TDP-43 mutations (Yu et al., 2020), it may also escape during the process of mitophagy/autophagy (Gkirtzimanaki et al., 2018; Oka et al., 2012).

In conclusion, we have shown that in a model human cell line, the absence of VPS13C results in late-endosome/lysosomal defects, as had been predicted by the localization of VPS13C at contacts between the ER and lysosomes and by the proposed role of VPS13C in mediating lipid exchange between these two organelles (Kumar et al., 2018; Leonzino et al., 2021). We have further discovered that these defects correlate with abnormally elevated STING signaling, most likely due to direct and indirect effects of the perturbation of lysosome function. An important question for future studies will be to determine the relevance of these findings in other model systems for PD pathophysiology, as they are not replicated in tissues of VPS13C^KO^ mice under basal conditions. Nonetheless, evidence in a model cell line of a link between loss-of-function of the PD gene VPS13C, lysosomal BMP biology, and the cGAS-STING pathway constitutes an intriguing new finding in the cell biology of PD.

## Materials and methods

### DNA plasmids

A plasmid containing codon-optimized cDNA encoding human VPS13C, with a Halo protein flanked by SacII restriction enzyme sites after amino acid residue 1,914, was generated by and purchased from GenScript Biotech. mCherry-Rab7a was obtained from Addgene (RRID:Addgene_61804). mCherry-Rab7a^Q67L^ and mCherry Rab7a^T22N^ were generated in our lab as previously reported (Guillen-Samander et al., 2019). Lamp1-mGFP was obtained from Addgene (RRID:Addgene_34831). GFP-VPS13C^βprop^ was generated in our lab as previously described (Kumar et al., 2018). TFEB-GFP was generated in the Ferguson lab as previously described (Roczniak-Ferguson et al., 2012; RRID:Addgene_38119). For CRISPR-mediated gene editing, candidate gRNAs against the human VPS13C genomic locus were identified using Benchling. gRNAs were ordered as complementary single-stranded oligonucleotides from Integrated DNA Technologies and then cloned into the PX459 plasmid (plasmid #62988; Addgene) using a one-step ligation protocol (Ran et al., 2013), and gRNAs were sequence verified using the U6 forward promoter. For CRISPR repair of the mutated VPS13C locus, gRNA was ordered (Integrated DNA Technologies) that incorporated a single nucleotide insertion present in one VPS13C allele in both of the VPS13C^KO^ clones. This was again cloned into the PX459 plasmid (RRID:Addgene_62988) using a one-step ligation protocol (Ran et al., 2013). To generate pMX-STING-GFP, STING-V1 plasmid was obtained from Addgene (RRID:Addgene_124262), and the STING coding sequence was amplified by PCR and ligated into pEGFP-N1 by using XhoI and SacII restriction sites. The STING-GFP coding sequence was then cut from the pEGFP-N1 backbone and ligated into a pMXs-IRES-Blasticidin Retroviral Vector backbone (RTV-016; Cell Biolabs) using XhoI and NotI restriction sites. Oligonucleotides used in this study are listed in Table S1. All oligonucleotides were purchased from Integrated DNA Technologies.

### Antibodies

Primary antibodies used: mouse α-tubulin (cat# T5168, RRID:AB_477579; Sigma-Aldrich), rabbit calnexin (cat# ADI-SPA-860-D, RRID:AB_2038898; Enzo Life Sciences), rabbit cathepsin D (cat# ab75852, RRID:AB_1523267; Abcam), rabbit CGAS (cat# 66546, RRID:AB_2799712; Cell Signaling Technology), mouse DNA (cat# CBL186, RRID:AB_9336; Millipore), rabbit EEA1 (cat#, PA1-063A, RRID:AB_2096819; Thermo Fisher Scientific), mouse GAPDH (cat# ADI-CSA-335, RRID:AB_10617247; Enzo Life Sciences), mouse GM130 (cat# 610822, RRID:AB_398141; BD Biosciences), rabbit HSP60 (cat# 12165, RRID:AB_2636980; Cell Signaling Technology), rabbit IRF3 (cat# 4302, RRID:AB_1904036; Cell Signaling Technology), mouse LAMP1 (cat# h4a3, RRID:AB_2296838; DSHB), rabbit Phospho-IRF3 (S396; cat# 4947, RRID:AB_823547; Cell Signaling Technology), rabbit Phospho-STING (S366; cat# 19781, RRID:AB_2737062; Cell Signaling Technology), rabbit Phospho-STING (S365; cat# 72971, RRID:AB_2799831; Cell Signaling Technology), rabbit Phospho-TBK1 (S172; cat# 14590, RRID:AB_2798527; Cell Signaling Technology), rabbit Rab7 (cat# 9367, RRID:AB_1904103; Cell Signaling Technology), rabbit STING (cat# 80231, RRID:AB_2799947; Cell Signaling Technology), rabbit TFEB (cat# 4240, RRID:AB_11220225; Cell Signaling Technology), rabbit TBK1 (cat# 3504, RRID:AB_2255663; Cell Signaling Technology), and rabbit VPS13C (custom, Proteintech).

### Cell culture and transfection

HeLa-M cells were cultured at 37°C in 5% CO_2_ and DMEM containing 10% FBS, 100 U/ml penicillin, 100 mg/ml streptomycin, and 2 mM L-glutamine (all from Gibco). For live-cell imaging experiments, cells were seeded on glass-bottomed dishes (MatTek) at a concentration of 35,000 cells per dish and transfected after 24 h using FuGene HD (Promega). For biochemical experiments, cells were plated at such a density as to be ∼90% confluent at the time of lysis. Transfection of siRNA was accomplished using Lipofectamine RNAiMax (Thermo Fisher Scientific). Introduction of cGAMP was accomplished using Lipofectamine RNAiMax (Thermo Fisher Scientific) as previously reported (Swanson et al., 2017). Transfection of HT-DNA was accomplished using Lipofectamine 2000 (Thermo Fisher Scientific). Plat-A cells for retroviral packaging were cultured at 37°C in 5% CO_2_ and DMEM containing 10% FBS, 100 U/ml penicillin, 100 mg/ml streptomycin, 2 mM L-glutamine, 1 μg/ml puromycin, and 10 μg/ml blasticidin.

For the i^3^Neuron cultures, we used an established WTC-11 human iPSC line (provided by Michael Ward, National Institute of Neurological Disorders and Stroke, Bethesda, MD) engineered to harbor a doxycycline-inducible NGN2 transgene expressed at the AAVS1 safe harbor locus to facilitate efficient differentiation into neurons (i^3^Neurons) with properties of layer 2/3 cortical glutamatergic pyramidal cells (Fernandopulle et al., 2018; Wang et al., 2017). Briefly, these iPSCs were grown on Matrigel-coated dishes in E8 media (Life Technologies). The protocol for differentiation into i^3^Neurons is described in detail by Fernandopulle et al. (2018) and has been used previously by our lab (Gowrishankar et al., 2021). A detailed protocol for HeLa cell cultures and transfections can be accessed on protocols.io at dx.doi.org/10.17504/protocols.io.36wgq41j3vk5/v1.

### Generation of stable STING-GFP cells using retrovirus

For retroviral packaging, 5 × 10^6^ Plat-A cells (Cell Biolabs) were plated on a 10-cm plate in media without antibiotics. The following day, cells were transfected with 9 µg of pMX-STING-GFP using Fugene HD (Promega). Retroviral supernatant was collected 72 h after transfection, supplemented with Polybrene (8 μg/ml), and passed through a 0.22-μm filter to remove cellular debris before being added to WT and VPS13C^KO^ HeLa cells. After 24 h, retroviral supernatant was removed and replaced with fresh complete DMEM. After an additional 24 h, HeLa cells were FACS sorted to enrich for GFP-positive cells. A detailed protocol can be accessed on protocols.io at dx.doi.org/10.17504/protocols.io.5jyl85xp7l2w/v1.

### Generation of CRISPR-KO and CRISPR-knock-in rescue cell lines

Early-passage HeLa-M cells were transfected with 1.5 µg of PX459 plasmid (plasmid #62988; Addgene) containing a sgRNA against VPS13C using Lipofectamine 2000 (Thermo Fisher Scientific). At 24 h after transfection, cells were selected in complete DMEM containing 2 μg/ml puromycin. At 48 and 72 h after transfection, medium was replaced with fresh puromycin-containing medium. After 3 d of puromycin selection, single clones were obtained using serial dilution and then screened by IB. Two clonal cell lines lacking VPS13C by IB were selected. Genomic DNA was extracted using a DNAeasy kit (Qiagen), and an ∼500-bp portion around the predicted CRISPR cut site was amplified by PCR and purified by NucleoSpin Gel and PCR Clean-up kit (Macherey-Nagel). This fragment was then ligated into an EGFP-N1 vector using Xho1 and Apa1 restriction sites and transformed into DH5-α–competent cells. After plating the transformation mix onto agar plates, >48 bacterial colonies per clone were submitted for Sanger sequencing to maximize the probability of sequencing all alleles. Frameshift mutations leading to premature stop codons were identified by Sanger sequencing.

To rescue VPS13C expression, we used CRISPR-mediated homology directed repair (HDR) using a single-stranded oligonucleotide DNA nucleotide (ssODN) as previously described (Okamoto et al., 2019). Briefly, a gRNA was synthesized that incorporated a single nucleotide insertion present in one VPS13C allele in both of the VPS13C^KO^ clones. An ssODN corresponding to the WT VPS13C sequence flanking the insertion was generated. Both VPS13C^KO^ clones were transfected with 1 μg of PX459 plasmid containing the gRNA against the mutant VPS13C allele and 100 pmol of ssODN. Puromycin selection and single clone selection were performed as above, and rescue of protein expression was confirmed by IB.

For CRISPR-Cas9–mediated gene editing of iPSCs, cells were harvested using accutase (Corning), and 1.5 million cells were resuspended in Mirus nucleofector solution and electroporated with 5 µg of PX458 plasmid (RRID:Addgene_48138) containing a gRNA targeted against the *VPS13C* gene using an Amaxa 2D nucleofector. Electroporated cells were then plated into one well of a 24-well plate, and GFP-positive cells were selected by FACS after 24 h. Cells were once again plated communally after sorting and serially diluted 72 h later to yield clonal populations for screening. Colonies were selected and screened using Western blot, followed by genomic sequencing of the VPS13C locus to confirm biallelic mutation. Detailed protocols for the creation of KO and rescue cell lines using CRISPR-Cas9 can be accessed on protocols.io at dx.doi.org/10.17504/protocols.io.eq2lynx5wvx9/v1.

### Light microscopy

#### Live-cell imaging

Before imaging, growth medium was removed and replaced with live-cell imaging solution (Life Technologies). All live-cell imaging was performed at 37°C in 5% CO_2_. Imaging was performed using an Andor Dragonfly spinning-disk confocal microscope equipped with a Plan Apochromat objective (63×, 1.4 NA, oil) and a Zyla scientific CMOS camera and acquired using Fusion software. For lysotracker experiments, cells were incubated in 50 nM LysoTracker Red DND-99 (Thermo Fisher Scientific) in complete DMEM for 30 min, washed twice with medium, and then imaged in live-cell imaging solution. A detailed protocol can be accessed on protocols.io at dx.doi.org/10.17504/protocols.io.36wgq41j3vk5/v1.

#### Immunofluorescence

WT and VPS13C^KO^ HeLa cells were plated on glass coverslips and fixed in a prewarmed (37°C) solution of 4% PFA in PBS for 15 min at room temperature, permeabilized with 0.1% (vol/vol) Triton X-100 in PBS for 10 min at room temperature, and blocked using filtered PBS containing 1% (wt/vol) BSA for 1 h at room temperature. Coverslips were then incubated with antibodies against DNA (CBL186, 1:150; EMD Millipore) and HSP60 (12165S, 1:1,000; CST) and diluted in filtered PBS containing 1% BSA at 4°C overnight, followed by 3× 5-min washes in PBS. Secondary antibodies (1:1,000, Alexa Fluor 488 and 555; Invitrogen) were incubated in PBS containing 1% BSA for 1 h at room temperature and removed by 3× 5-min washes in PBS. Finally, coverslips were mounted onto slides using ProLong Gold Antifade Mountant with DAPI (P36935; Thermo Fisher Scientific) and allowed to cure overnight at room temperature before imaging. A detailed protocol for immunofluorescent staining can be accessed on protocols.io at dx.doi.org/10.17504/protocols.io.14egn741mv5d/v1.

#### Image processing and analysis

Fluorescence images were processed using Fiji software (v2.0.0-rc-69/1.52i, https://fiji.sc, RRID:SCR_002285). For quantification of lysotracker images (Fig. 1 E), cells were outlined manually, and their average fluorescence intensity was measured. Intensities were then normalized such that the average intensity of the WT cells was 1. For quantification of TFEB-GFP nuclear/cytoplasmic ratio (Fig. S1 D), masks were drawn manually around the border, and nucleis of individual cells and mean intensities were measured. For quantification of stable STING-GFP cells, cells with a reticular ER or vesicular pattern were counted manually, and the percentage of cells with punctate STING-GFP distribution was calculated.

### IB

Cultured cells were lysed on ice by repeat pipetting in radioimmunoprecipitation assay (RIPA) buffer (150 mM NaCl, 10 mM Tris, 0.5 mM EDTA, and 0.5% NP-40) supplemented with Protease Inhibitor Cocktail (Roche) and PhosStop phosphatase inhibitor (Roche). Mouse tissue samples were submerged in ice cold RIPA buffer supplemented with Protease Inhibitor Cocktail and PhosStop phosphatase inhibitor and homogenized using Potter-Elvehjem homogenizers, followed by repeat pipetting. Samples were then centrifuged at 15,000 *g* for 10 min at 4°C, and the postnuclear supernatant was collected. Total protein content was then measured by Pierce BCA assay (Thermo Fisher Scientific). Samples were prepared for IB at equal protein concentrations in 3× Laemmli buffer (188 mM Tris-HCl, 3% SDS, 30% glycerol, 0.01% bromophenol blue, and 15% β-mercaptoethanol) and denatured at 95°C for 5 min. Proteins were separated on Mini PROTEAN TGX 4–20% Tris-glycine gels (Bio-Rad) before transfer to nitrocellulose membranes at 4°C for 1 h at 120 V in transfer buffer containing 25 mM Tris, 192 mM glycine, and 20% methanol in milliQ water. Total protein was visualized using Ponceau stain and blocked in 5% milk in TBS containing 0.1% Tween-20 (TBST) for 1 h. Membranes were then incubated with primary antibodies in 2.5% milk in TBST overnight at 4°C. The next day, membranes were washed 3× in TBST and then incubated with secondary antibodies conjugated to IRdye 800CW or IRdye 680CW (1:10,000; Licor) in 2.5% milk in TBST at 22°C for 1 h, washed 3× with TBST and 3× with TBS, and then imaged using a Licor Odyssey Infrared Imager.

For VPS13C IB, postnuclear supernatant was collected as above. Samples for IB were prepared using NuPAGE LDS Sample Buffer and Reducing Agent (Thermo Fisher Scientific) and incubated for 10 min at 70°C. Proteins samples were separated on NuPage Tris-Acetate 3–8% gels and then transferred onto nitrocellulose membranes at 4°C overnight at 0.5 mA in NuPAGE transfer buffer containing 20% methanol. Membranes were blocked as above and incubated with primary antibody against VPS13C (custom, 1:400; Proteintech) in 2.5% milk in TBST for 2 h at 22°C. Membranes were incubated with secondary antibodies, washed, and imaged as above. A detailed protocol can be accessed on protocols.io at dx.doi.org/10.17504/protocols.io.bp2l6be9zgqe/v1.

### Synthesis of colloidal dextran-conjugated SPIONs

SPIONs were synthesized according to the following protocol: 10 ml of aqueous 1.2 M FeCl_2_ and 10 ml of 1.8 M FeCl_3_ were combined in a 500-ml glass beaker with magnetic stirring, followed by slow addition of 10 ml of 30% NH_4_OH. This mixture was stirred for 5 min; during that time, a dark brown sludge formed. The beaker was then placed on a strong magnet to allow the particles to migrate toward the magnet. The supernatant was removed, and the particles were resuspended in 100 ml water, followed by repeated separation on the magnet. This step was repeated two more times. Particles were then resuspended in 80 ml of 0.3 M HCl and stirred for 30 min, followed by the addition of 4 g of dextran and another 30 min of stirring. Particles were transferred into dialysis bags and dialyzed for 48 h in milliQ water with water changes approximately every 12 h. The resulting mixture was then centrifuged at 19,000 *g* for 15 min to remove large aggregates. Supernatant containing nanoparticles was stored at 4°C. Detailed protocols for synthesis of SPIONs can be accessed on protocols.io at dx.doi.org/10.17504/protocols.io.eq2lyn69pvx9/v1.

### Purification of lysosomes with dextran-conjugated SPIONs

WT and VPS13C^KO^ HeLa cells were plated on 4 × 15-cm plates at 3.5 × 10^6^ cells per dish. The next day, the culture medium (DMEM) was exchanged for fresh DMEM containing 10 mM Hepes and 10% SPION solution by volume for 4 h (pulse). The medium was then changed back to fresh DMEM, and after 15 h (chase), the cells were washed twice with PBS and then scraped into 5 ml of PBS on ice. Cells were then centrifuged at 1,000 rpm for 10 min at 4°C. PBS was removed, and the cell pellet was resuspended in 3 ml homogenization buffer (HB; 5 mM Tris, 250 mM sucrose, and 1 mM EGTA, pH 7.4) supplemented with protease inhibitor cocktail (Roche) immediately before use and passed through a manual cell homogenizer (Isobiotec; 10 cycles, 10-µm clearance) to generate a total cell lysate. The lysate was centrifuged at 800 *g* for 10 min at 4°C, and the supernatant was loaded onto a magnetic LS column (Miltenyi Biotec) pre-equilibrated with 1 ml of HB. The column was washed with 5 ml HB, removed from the magnetic rack, and eluted with three successive aliquots of 1 ml HB forced through with positive pressure. The eluate was then centrifuged at 55,000 rpm for 1 h at 4°C to pellet the lysosome fraction, and the resulting pellet was resuspended in 200 µl of MS-grade water (Thermo Fisher Scientific) and flash frozen. Fluorescence images of nanoparticles were obtained using FluoreMAG A ferrofluid (Liquids Research) with a 4-h pulse and 15-h chase. Detailed protocols for the purification of lysosomes with dextran-conjugated SPIONs can be accessed on protocols.io at dx.doi.org/10.17504/protocols.io.bp2l61dr1vqe/v1.

### Lipid extraction for MS lipidomics

MS-based lipid analysis was performed by Lipotype as described (Sampaio et al., 2011). Lipids were extracted using a two-step chloroform/methanol procedure (Ejsing et al., 2009). Samples were spiked with an internal lipid standard mixture containing: cardiolipin 16:1/15:0/15:0/15:0 (CL), ceramide 18:1;2/17:0 (Cer), diacylglycerol 17:0/17:0 (DAG), hexosylceramide 18:1;2/12:0 (HexCer), lyso-phosphatidate 17:0 (LPA), lyso-phosphatidylcholine 12:0 (LPC), lyso-phosphatidylethanolamine 17:1 (LPE), lyso-phosphatidylglycerol 17:1 (LPG), lyso-phosphatidylinositol 17:1 (LPI), lyso-phosphatidylserine 17:1 (LPS), phosphatidate 17:0/17:0 (PA), phosphatidylcholine 17:0/17:0 (PC), phosphatidylethanolamine 17:0/17:0 (PE), phosphatidylglycerol 17:0/17:0 (PG), phosphatidylinositol 16:0/16:0 (PI), phosphatidylserine 17:0/17:0 (PS), cholesterol ester 20:0 (CE), sphingomyelin 18:1;2/12:0;0 (SM), and triacylglycerol 17:0/17:0/17:0 (TAG). After extraction, the organic phase was transferred to an infusion plate and dried in a speed vacuum concentrator. The first-step dry extract was resuspended in 7.5 mM ammonium acetate in chloroform/methanol/propanol (1:2:4, vol/vol/vol), and the second-step dry extract, in a 33% ethanol solution of methylamine in chloroform/methanol (0.003:5:1; vol/vol/vol). All liquid handling steps were performed using Hamilton Robotics STARlet robotic platform with the Anti Droplet Control feature for organic solvents pipetting.

### MS data acquisition

Samples were analyzed by direct infusion on a QExactive mass spectrometer (Thermo Fisher Scientific) equipped with a TriVersa NanoMate ion source (Advion Biosciences). Samples were analyzed in both positive and negative ion modes with a resolution of R_m/z=200_ = 280,000 for MS and R_m/z=200_ = 17,500 for MSMS experiments, in a single acquisition. MSMS was triggered by an inclusion list encompassing corresponding MS mass ranges scanned in 1-dalton increments (Surma et al., 2015). Both MS and MSMS data were combined to monitor CE, DAG, and TAG ions as ammonium adducts; PC and PC O- as acetate adducts; and CL, PA, PE, PE O-, PG, PI, and PS as deprotonated anions. MS only was used to monitor LPA, LPE, LPE O-, LPI, and LPS as deprotonated anions and Cer, HexCer, SM, LPC, and LPC O- as acetate adducts.

### Data analysis and postprocessing

Data were analyzed by Lipotype with in-house-developed lipid identification software based on LipidXplorer (Herzog et al., 2012; Herzog et al., 2011). Data postprocessing and normalization were performed using an in-house-developed data management system. Only lipid identifications with a signal-to-noise ratio >5 and signal intensity fivefold higher than in corresponding blank samples were considered for further data analysis. Lipidomics datasets are available at https://doi.org/10.5281/zenodo.6416363.

### Measurement of di-22:6-BMP and di-18:1-BMP

Targeted high-resolution UPLC-MS/MS was used to accurately quantitate total di-22:6-BMP (the sum of its three isoforms). Total di-18:1-BMP was measured as well. Quantitation was performed by Nextcea using authentic di-22:6-BMP and di-18:1-BMP reference standards. Di-14:0-BMP was used as an internal standard.

### Depletion of mtDNA

HeLa cells were treated with 2 μg/ml EtBr in DMEM for 8 d. Medium was replaced every 2 d, and cells were passaged on day 4. On day 8, cells were lysed, and total DNA, RNA, and protein were collected. Depletion of mtDNA and levels of ISG transcripts were assayed using qPCR as described below. Detailed protocols for depletion of mtDNA can be accessed on protocols.io at dx.doi.org/10.17504/protocols.io.rm7vzy6e5lx1/v1.

### qPCR

RNA from cultured cells was extracted using the RNeasy Plus Micro RNA extraction kit followed by reverse transcription using iScript cDNA synthesis Kit (Bio-Rad). RNA from mouse brain tissue was extracted using TRIzol reagent and an RNeasy Lipid Tissue Mini kit according to commercial protocol. Equal amounts of DNA and corresponding primers (Table S1) were used for qPCR using SYBR Green Master Mix. For each biological sample, two technical replicates were performed. Mean values were normalized against the β-actin threshold cycle (Ct) value to calculate ΔCt. The ΔCt of each sample was compared to the ΔCt of the WT sample to generate the ΔΔCt value. Relative expression was then analyzed using the 2^−ΔΔCt^ method, and the relative fold-change was plotted with the WT samples given a value of 1. To quantify mtDNA, DNA was extracted from total cell lysate and cytosolic fractions using DNeasy kit (Qiagen). DNA samples were each diluted 1:10, and corresponding primers (Table S1) were used for qPCR using SYBR Green Master Mix. Two technical replicates were performed for each biological sample, and mean Ct values of mtDNA amplicons form cytosolic fractions were normalized against the corresponding total cell lysate hB2M (nuclear DNA control) Ct value. Relative copy number was determined by the 2^−ΔΔCt^ method, and the WT mtDNA abundance was given a value of 1. A detailed protocol for qPCR can be accessed on protocols.io at dx.doi.org/10.17504/protocols.io.14egnz12qg5d/v1.

### Fractionation of cytosol by centrifugation

Cytoplasmic buffer (CB; 150 mM NaCl, 50 mM Hepes, and 1 mg/ml digitonin, pH 7.4) and lysis buffer (CB + 1% SDS supplemented with Protease Inhibitor Cocktail [Roche]) were prepared. HeLa cells were plated in 15-cm plates, 3.5 × 10^6^ per plate. After 24 h, cells were trypsinized and centrifuged at 1,500 rpm for 5 min at 22°C. Cells were resuspended in PBS and counted. For each genotype, 5 × 10^6^ cells were collected and centrifuged at 1,500 rpm for 5 min at 22°C. Cells were resuspended in 1 ml PBS, and 50 µl was transferred to a prechilled Eppendorf tube (WCE) and kept on ice. The remaining 950 µl was transferred to a prechilled Eppendorf tube and centrifuged at 4,500 RPM for 5 min at 4°C. The supernatant was removed, and the cells were resuspended in CB and rotated for 10 min at 4°C. Sample was centrifuged at 980 *g* for 3 min at 4°C. The supernatant was transferred to a new Eppendorf, and the pellet was flash frozen for analysis. The supernatant was centrifuged at 17,000 *g* for 10 min at 4°C. The supernatant was again collected. DNA was collected from these samples using a DNeasy kit (Qiagen). A detailed protocol can be accessed on protocols.io at dx.doi.org/10.17504/protocols.io.14egnz12yg5d/v1.

### Vps13c^−/−^ mice

All animal studies were conducted in compliance with guidelines from the US Department of Health and Human Services Guide for the Care and Use of Laboratory Animals under Yale Institutional Animal Care and Use Committee protocol 2021_07422. Cryopreserved sperm from *Vps13c*^tm1a^ mice was obtained from the European Mutant Mouse Archive (http://www.informatics.jax.org/allele/MGI:5548822) and sequentially bred with FLP recombinase and β-actin-Cre transgenic mice to generate the *Vps13c*^tm1d^ (*Vps13c*^−/−^) allele. Loss of function of *Vps13c* was confirmed both at the genome level by Sanger sequencing using Transnetyx and at the protein level by IB. For protein and RNA extraction, mice were euthanized in a CO_2_ chamber, and brains were dissected and placed immediately on ice before being processed as described in the IB and qPCR sections.

### Balance beam assay

Mice were placed on one end of a 50 × 1-cm narrow cylindrical dowel suspended 20 cm above a padded surface. On the other end of the beam was placed a box containing bedding material from each mouse’s cage. The time which each animal took to traverse the beam the first time was recorded, as was the total number of runs each mouse was able to perform in 1 min. A detailed protocol for the balance beam assay can be accessed on protocols.io at dx.doi.org/10.17504/protocols.io.bp2l618edvqe/v1.

### Rotarod assay

Mice were placed on a four-lane Rotarod apparatus (Columbus Instruments), which increased in speed from 4 to 40 rpm over 5 min. The length of time before falling was recorded for each animal. At total of four trials were conducted per animal, and the results were averaged. A detailed protocol for the Rotarod assay can be accessed on protocols.io at dx.doi.org/10.17504/protocols.io.j8nlkkd1dl5r/v1.

### BMDM isolation and culture

For BMDM isolation, 11–12-wk-old male WT and *Vps13c*^−/−^ mice were euthanized in a CO_2_ chamber. Femurs were collected, and bone marrow cavities were flushed with 5 ml ice-cold PBS. After centrifugation to collect bone marrow cells, the pellet was resuspended and plated onto plastic Petri dishes, and cells were differentiated for 6 d in the culture medium containing DMEMF12 (Gibco) base medium, 20% FBS (Gibco), 20% L929 conditioned medium, 1% penicillin-streptomycin (Gibco), and 1% glutaMAX (Gibco).

### Mouse fibroblast isolation and culture

P0 pups from a *Vps13c*^+/−^/*Vps13c*^+/−^ breeding pair were collected and decapitated, and a piece of the tail was collected for genotyping. The bodies were soaked in 70% ethanol and washed with sterile PBS. The skin was then removed and placed in a 10-cm cell culture dish using a pair of forceps. 2–3 ml of ice-cold 0.25% trypsin-EDTA was added to the dish, and the skin tissue was finely chopped using sterile forceps and a sterile surgical blade. Tissue was transferred to a 15-ml Falcon tube, and ice-cold 0.25% trypsin-EDTA was added to bring the total volume to 3 ml before letting the tube stand at 4°C overnight. The next morning, most of the trypsin solution was aspirated, leaving approximately two volumes of the tissue. The tube was then incubated for 30 min in a 37°C water bath. 8 ml of fibroblast culture medium (DMEM containing 10% FBS, 100 U/ml penicillin, 100 mg/ml streptomycin, 1 mM sodium pyruvate, and 100 mM β-mercaptoethanol) was added to each tube and pipetted vigorously to break up digested tissues and form a cell suspension. Larger chunks of tissue were allowed to sediment to the bottom of the tube by gravity for 1 min. The supernatant was then transferred to another tube. 7 ml fibroblast medium was re-added to the remaining tissue clumps and allowed to sediment again, and the supernatant was added to the previously collected cell suspension. The suspension was then plated in 10-cm cell culture dishes. Primary fibroblasts were expanded and frozen.

### Statistical analyses

GraphPad Prism (v8.0.1, http://www.graphpad.com/, RRID:SCR_002798) was used for statistical comparison of live cell imaging, IB densitometry, qPCR, BMP measurement, and motor assays. Data distribution was assumed to be normal but was not formally tested. Two-sided Student’s *t* tests were used to assess significant differences between groups. Time course data were compared using two-way ANOVA followed by false discovery rate (FDR) multiple comparisons testing. Statistical analysis for lipidomic data was performed using RStudio (R v3.5.3, https://www.rstudio.com, RRID:SCR_000432). Groups were compared using Student’s *t* test followed by adjustment for multiple comparisons using Benjamini–Hochberg methodology to control FDR. q Values were determined using the R package “q value,” (http://www.bioconductor.org/packages/release/bioc/html/qvalue.html, RRID:SCR_001073), and a significance threshold of 0.05 was used. R codes used in this manuscript can be found at the following DOI: https://zenodo.org/badge/latestdoi/482411370.

### Online supplemental material

Fig. S1 shows supporting data for Fig. 1. Fig. S2 shows lipidomics of whole-cell and purified lysosomes. Fig. S3 shows control experiments for Fig. 4 and shows that VPS13C^KO^ cells have normal mtDNA nucleoid morphology. Fig. S4 shows supporting data for Figs. 5 and 6 as well as EGFR and LC3 degradation. Fig. S5 shows data from *Vps13c*^*−/−*^ mice, which do not display motor defects or STING activation. Video 1 shows changes to STING-GFP localization induced by treatment with cGAMP. Table S1 shows sequences of oligonucleotides used in this study.

## Supplementary Material

Table S1 lists oligonucleotides used in this study

SourceData F1 is the source file for Fig. 1.

SourceData F3 is the source file for Fig. 3.

SourceData F4 is the source file for Fig. 4.

SourceData F5 is the source file for Fig. 5.

SourceData F6 is the source file for Fig. 6.

SourceData FS1 is the source file for Fig. S1.

SourceData FS3 is the source file for Fig. S3.

SourceData FS4 is the source file for Fig. S4.

SourceData FS5 is the source file for Fig. S5.
