# Supplementary figures and images for: ER-lysosome lipid transfer protein VPS13C/PARK23 prevents aberrant mtDNA-dependent STING signaling
Source: J Cell Biol. 2022 Jun 3;221(7):e202106046. doi: 10.1083/jcb.202106046 (PMC9170524; doi:10.1083/jcb.202106046)

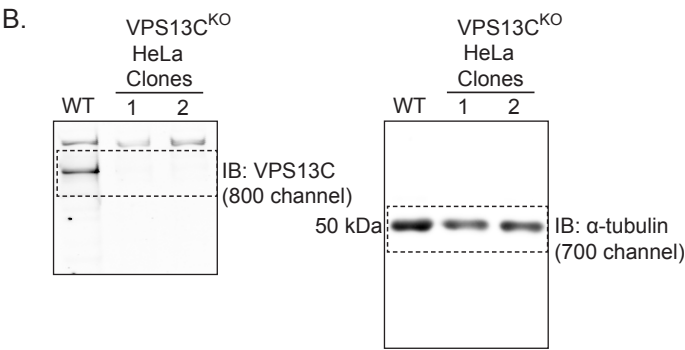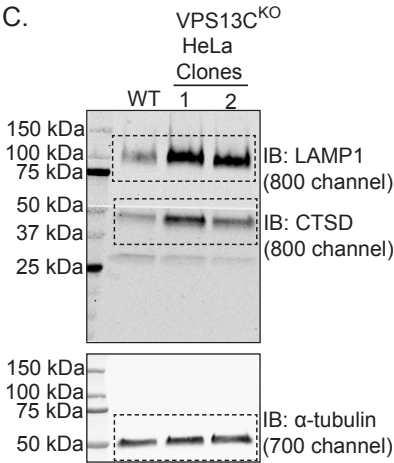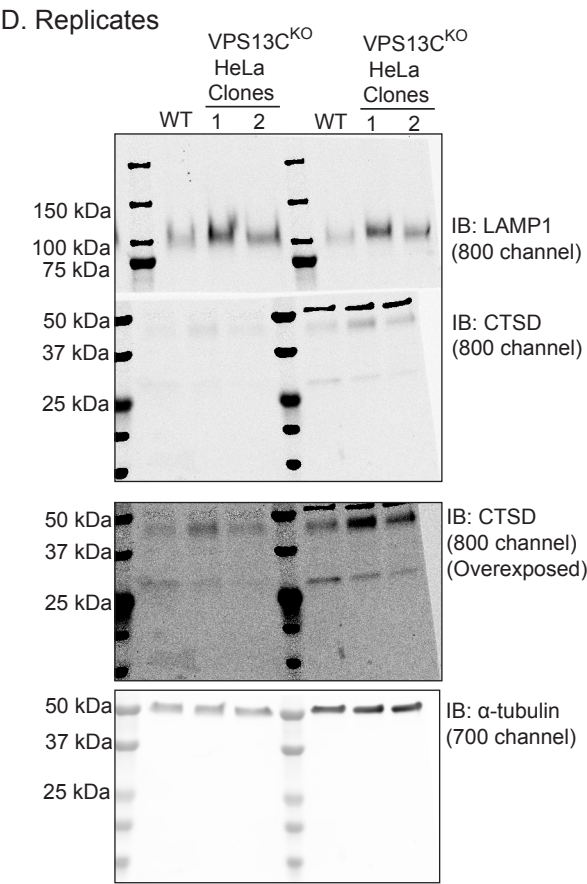

**H. Replicates**

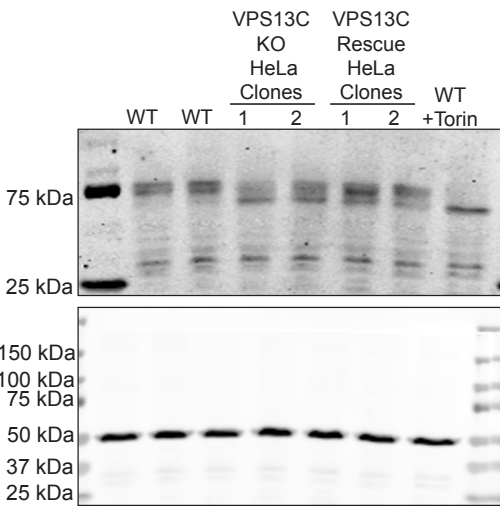

**G.**

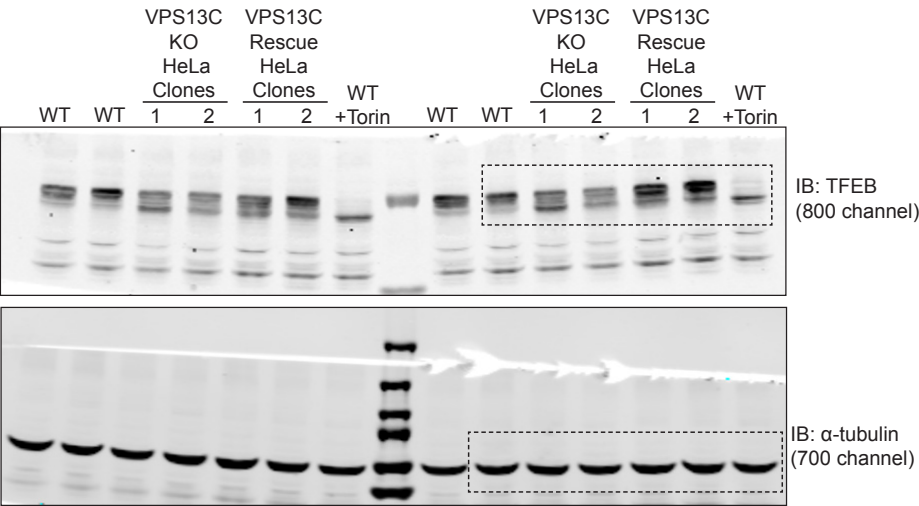

Supplement: SourceData F1 — is the source file for Fig. 1. [file JCB_202106046_SourceDataF1.pdf]

D.

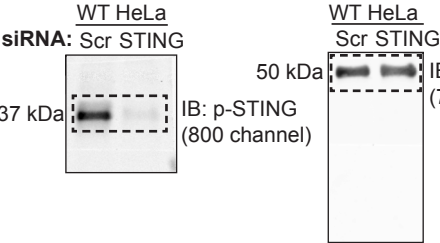

G.

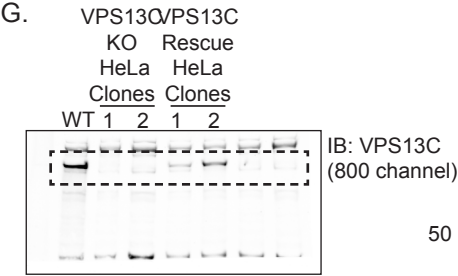

F.

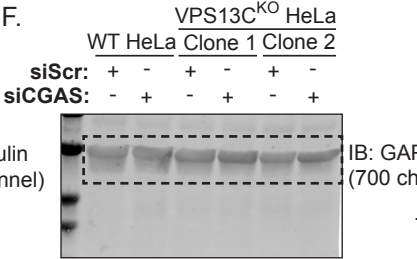

F.

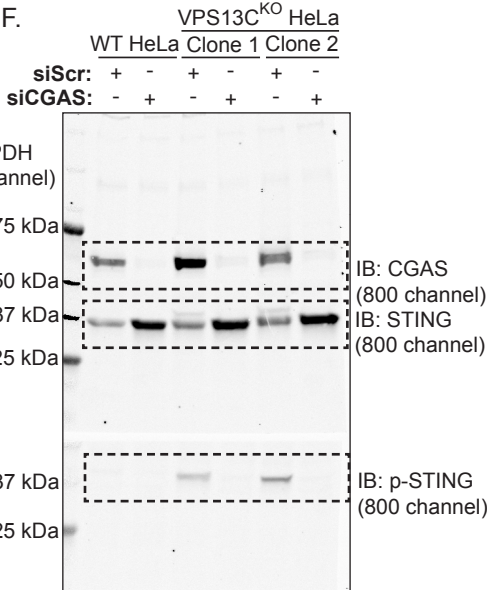

E.

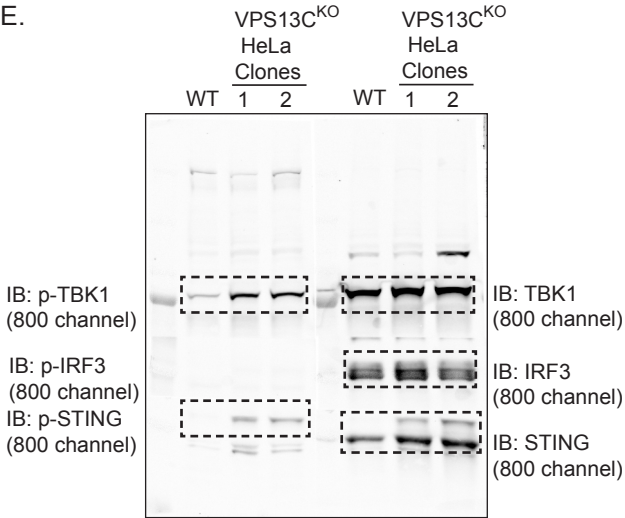

E.

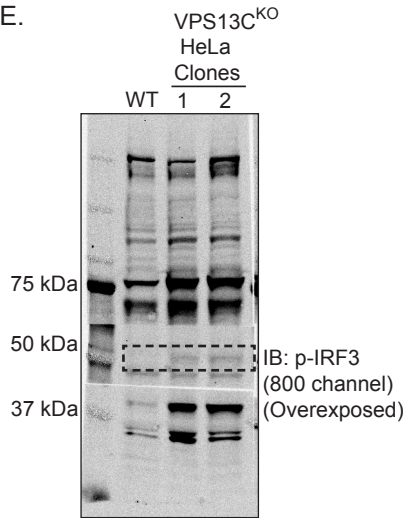

E.

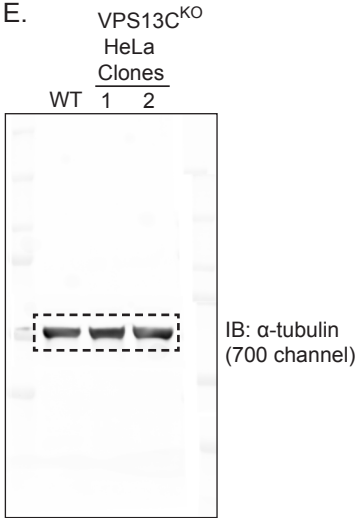

G.

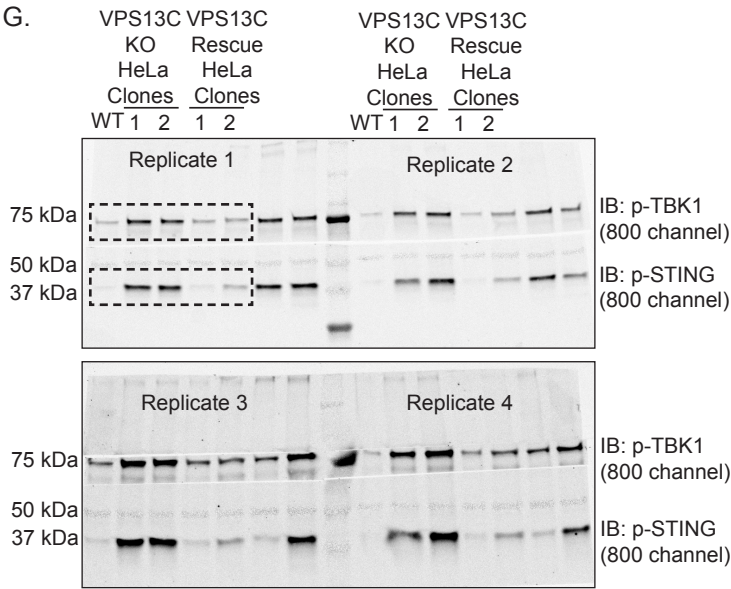

G.

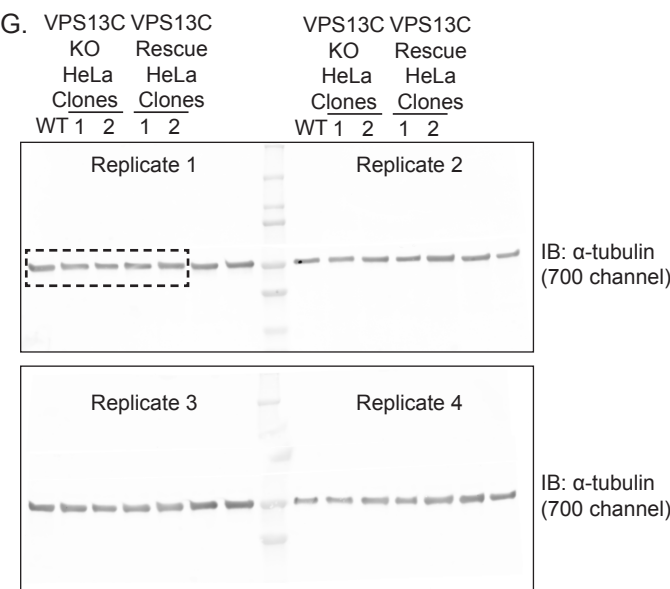

Supplement: SourceData F3 — is the source file for Fig. 3. [file JCB_202106046_SourceDataF3.pdf]

B.

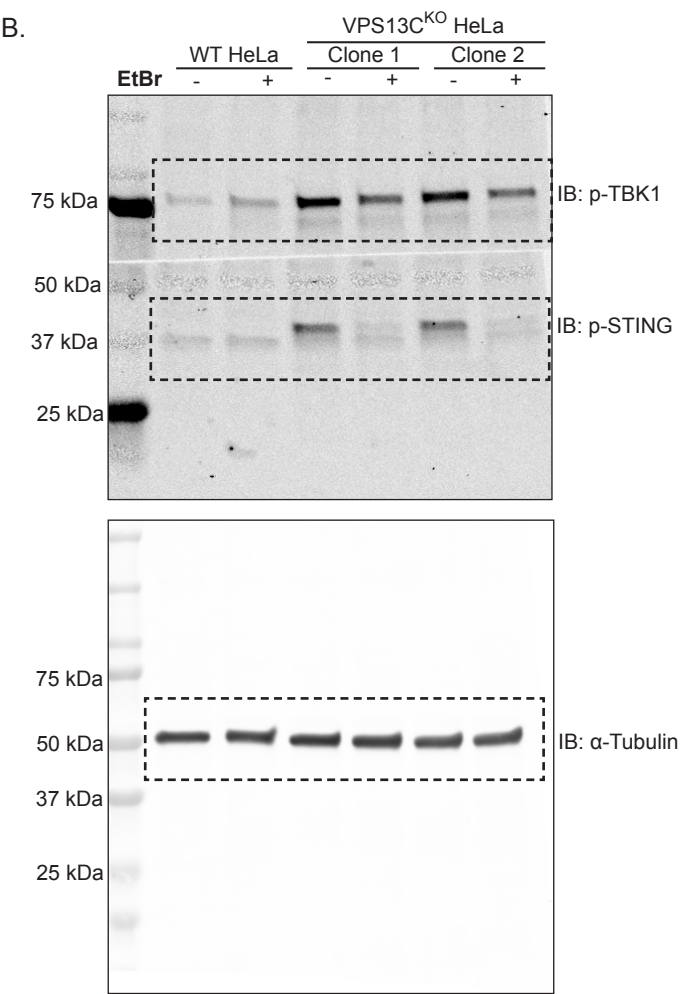

Supplement: SourceData F4 — is the source file for Fig. 4. [file JCB_202106046_SourceDataF4.pdf]

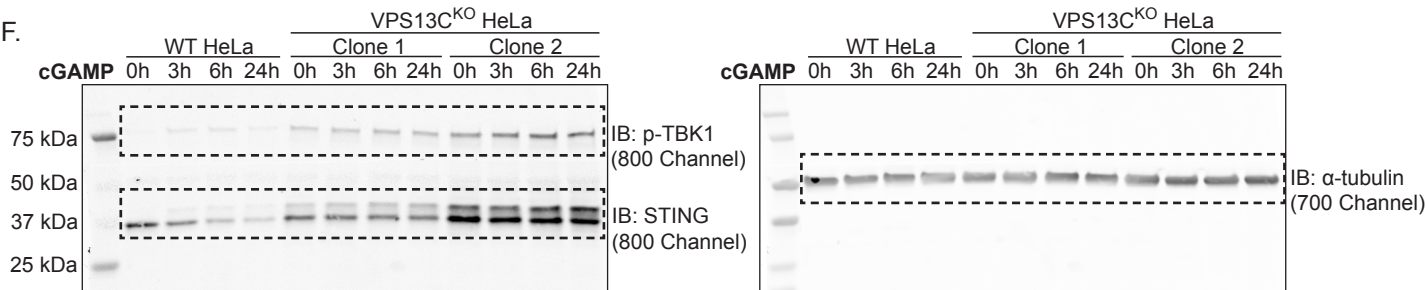

G. Replicates

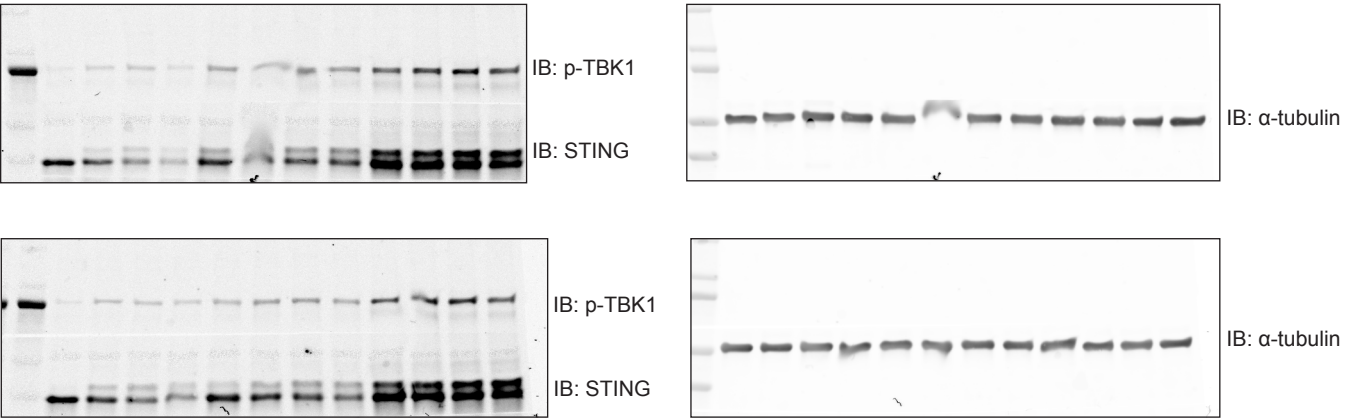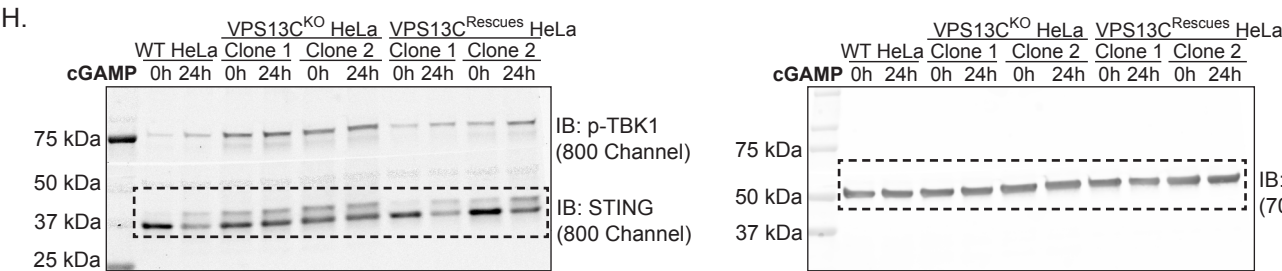

I. Replicates

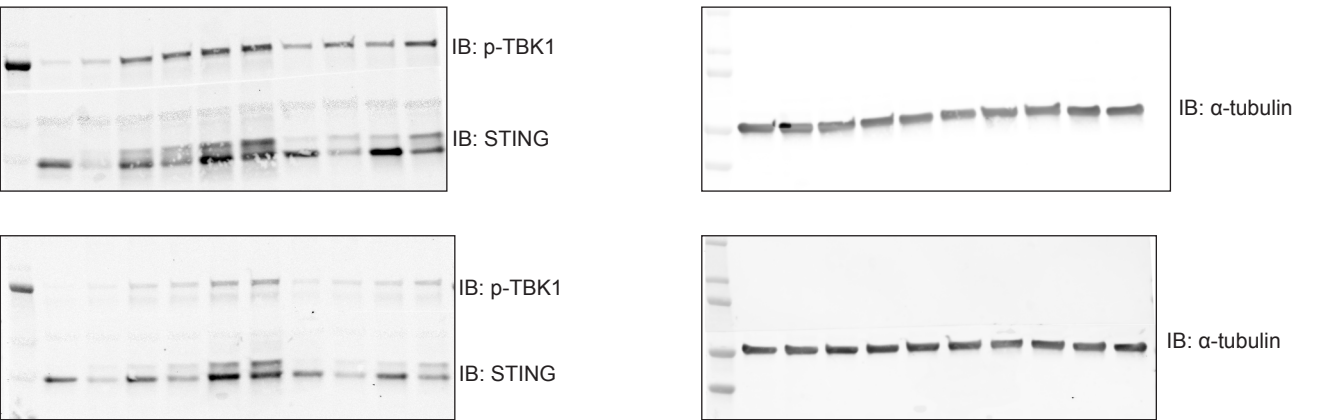

Supplement: SourceData F5 — is the source file for Fig. 5. [file JCB_202106046_SourceDataF5.pdf]

SourceDataF6  
D,F. (same blot as Figure S4F)

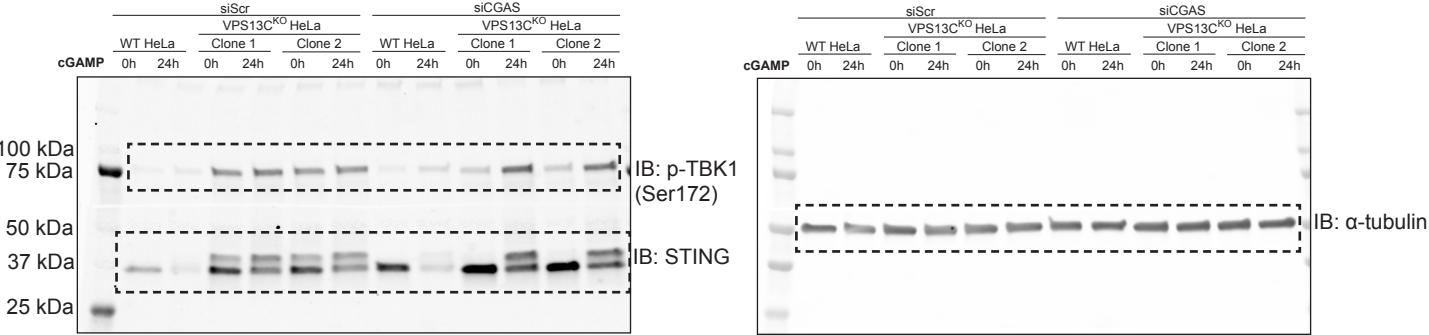

E,G. Replicates

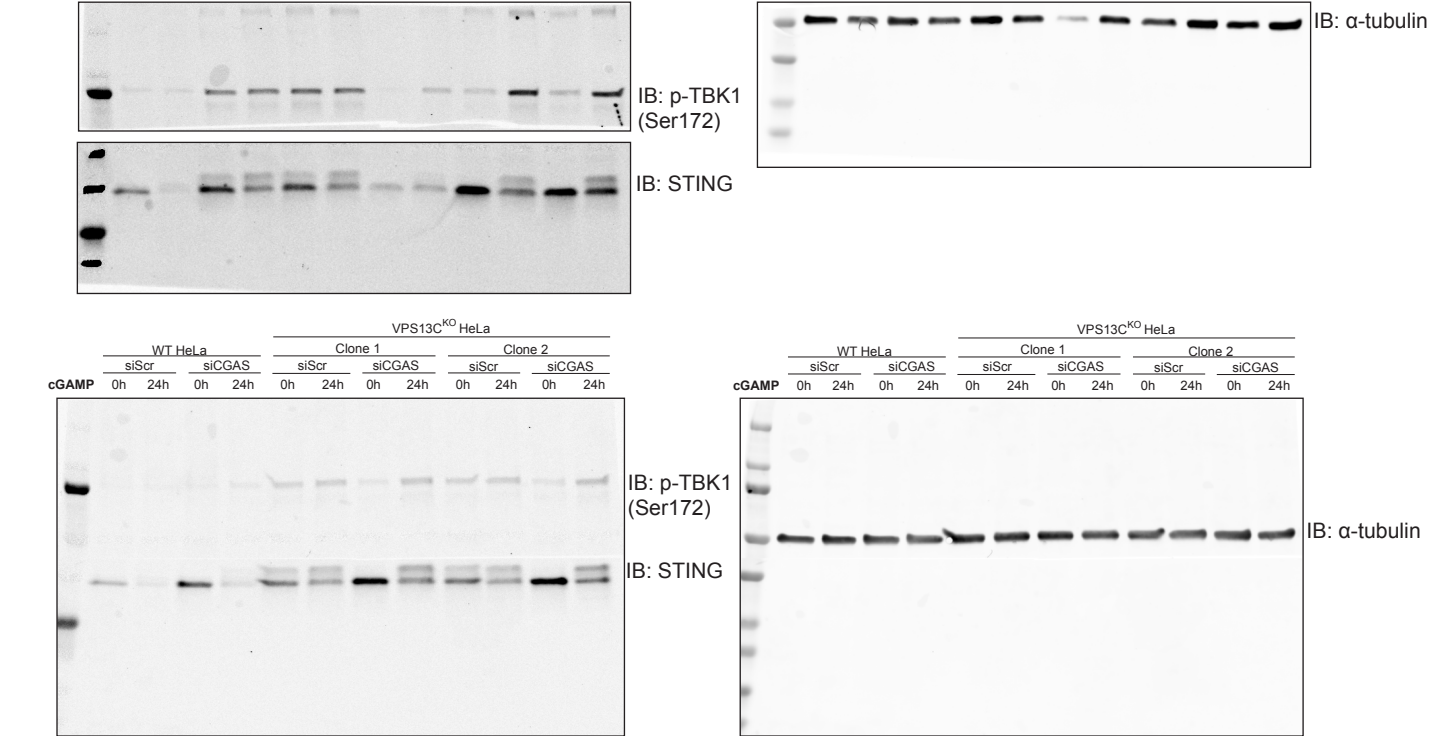

Supplement: SourceData F6 — is the source file for Fig. 6. [file JCB_202106046_SourceDataF6.pdf]

E.

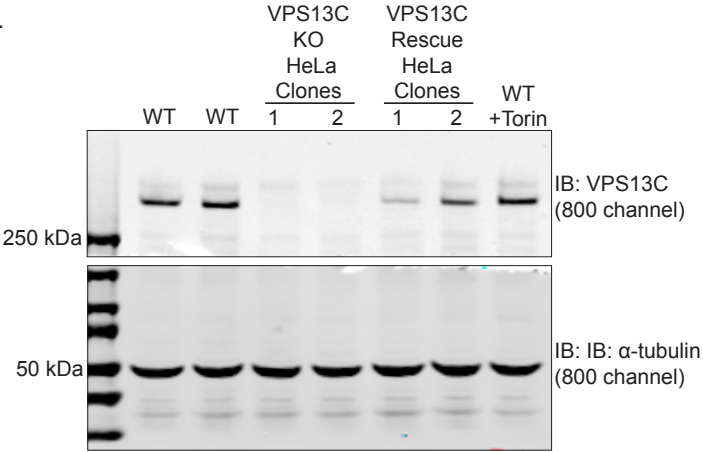

Supplement: SourceData FS1 — is the source file for Fig. S1. [file JCB_202106046_SourceDataFS1.pdf]

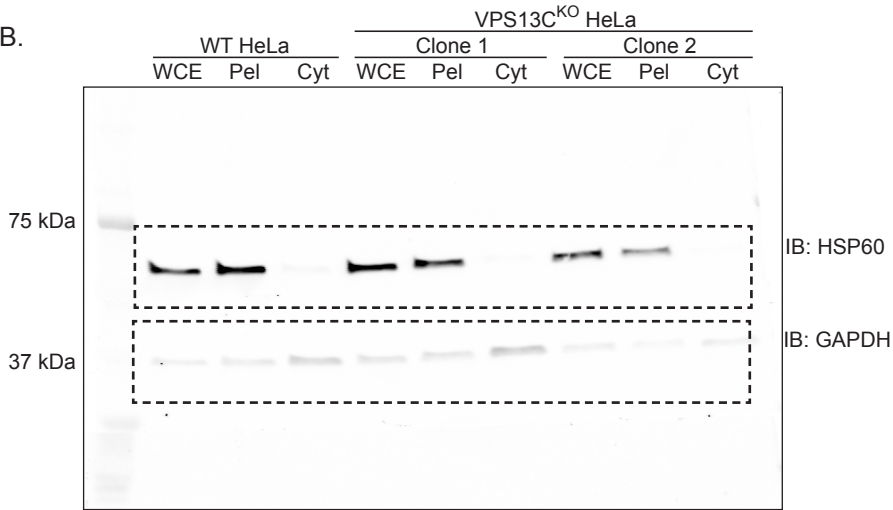

Supplement: SourceData FS3 — is the source file for Fig. S3. [file JCB_202106046_SourceDataFS3.pdf]

H.

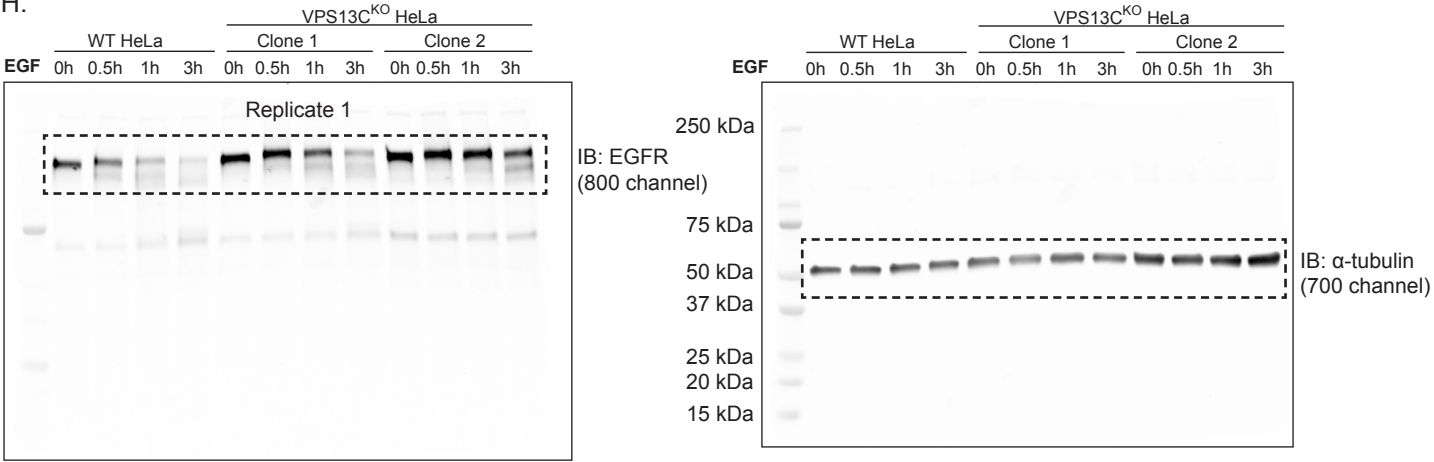

I. Replicates

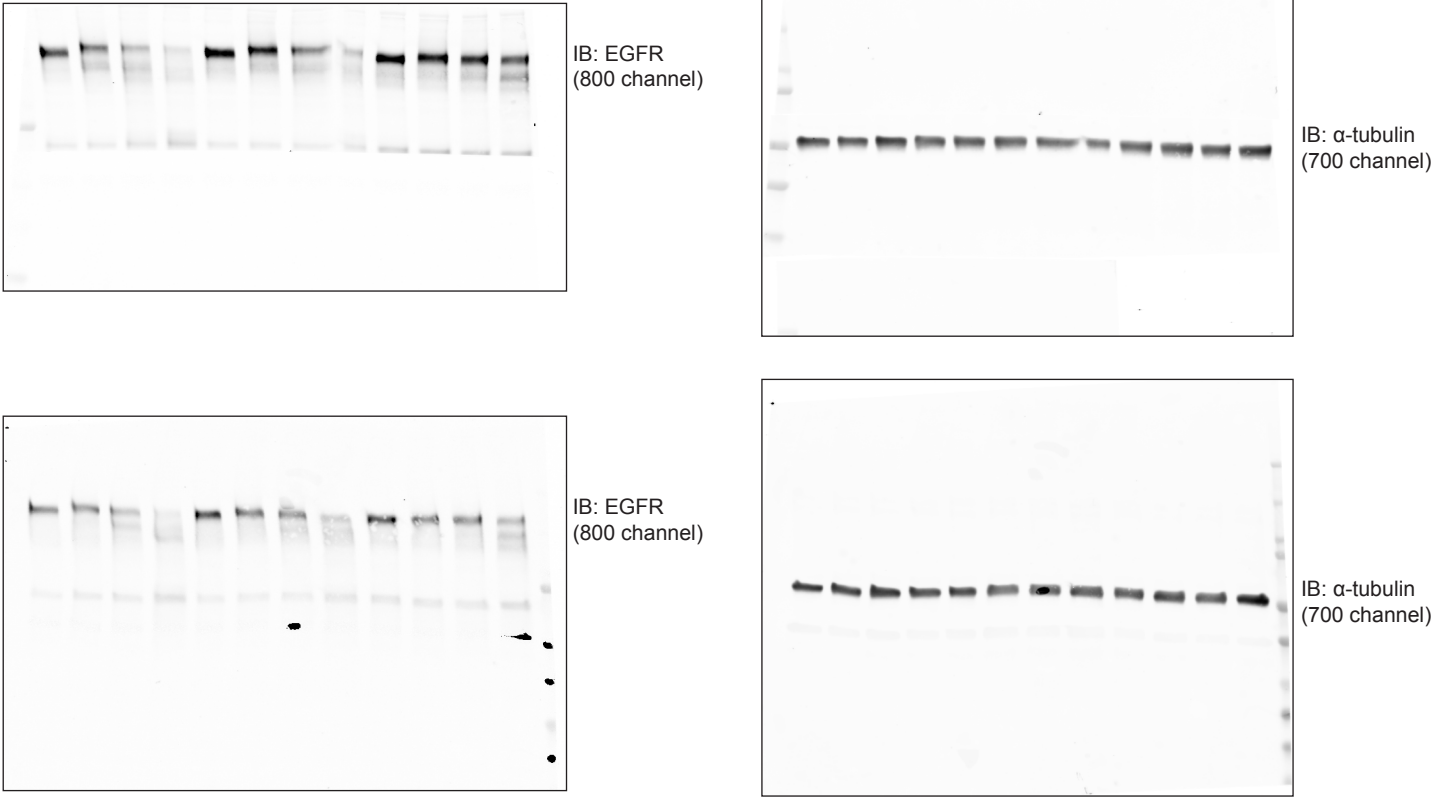

J.

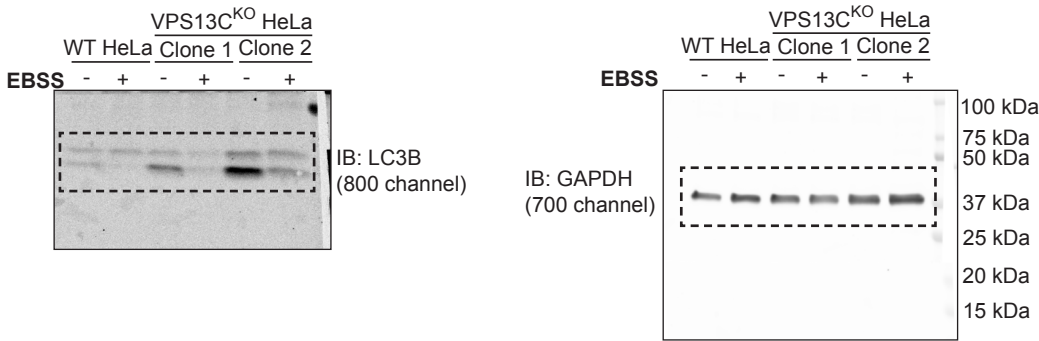

Supplement: SourceData FS4 — is the source file for Fig. S4. [file JCB_202106046_SourceDataFS4.pdf]

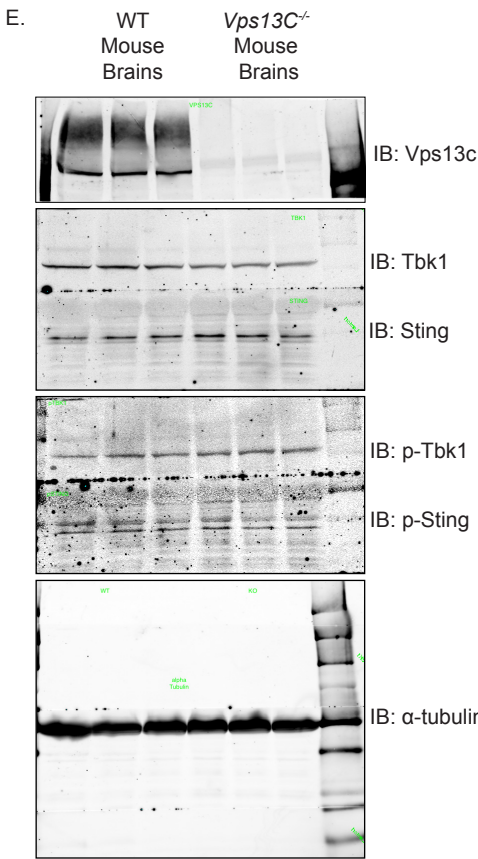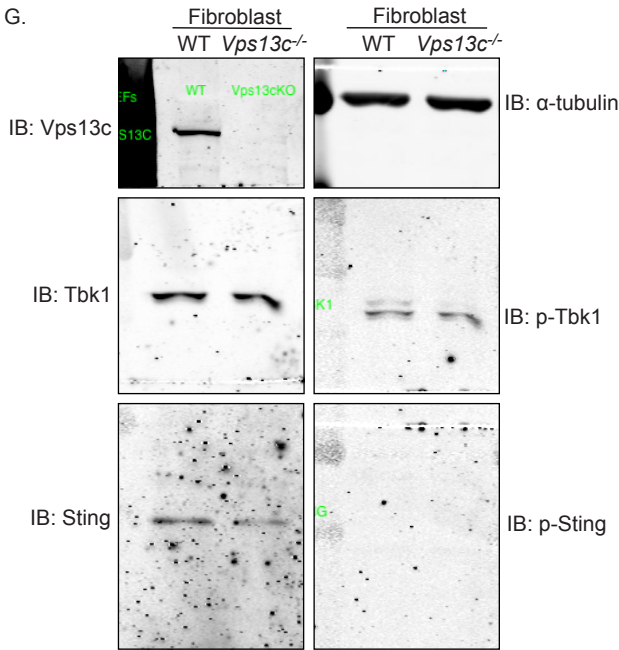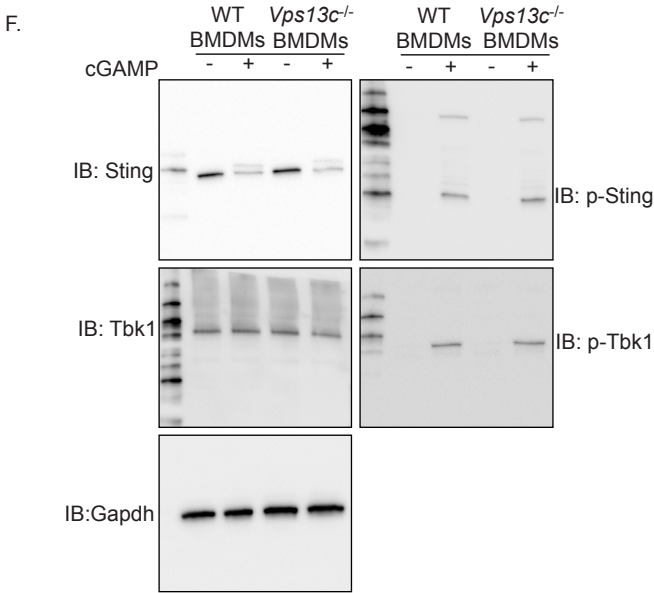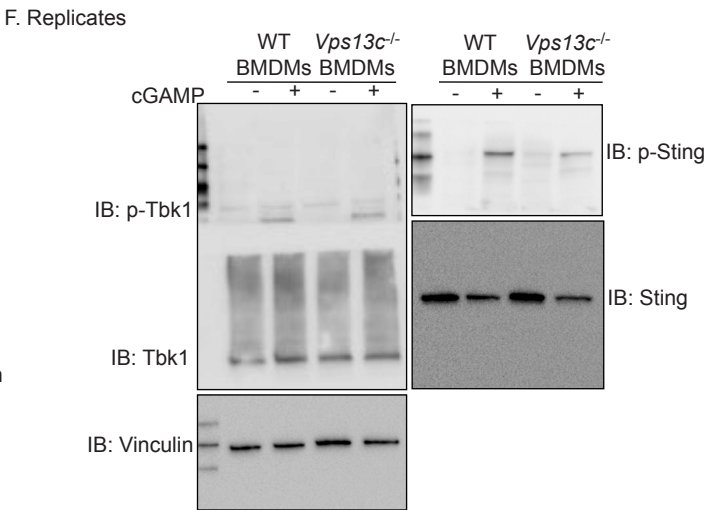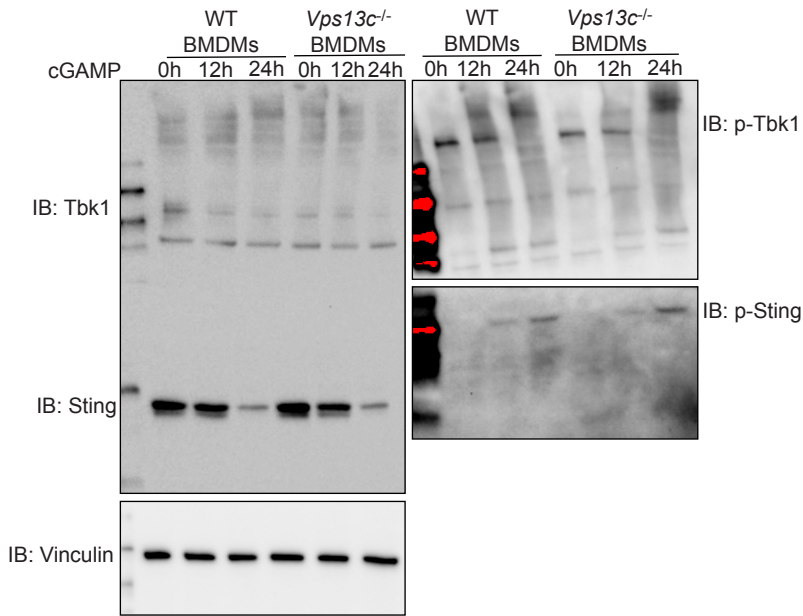

Supplement: SourceData FS5 — is the source file for Fig. S5. [file JCB_202106046_SourceDataFS5.pdf]
